# Clinically-applicable prediction of hospital stay and patient similarity retrieval in paediatric cardiology using machine learning

**DOI:** 10.1038/s41467-026-73021-3

**Published:** 2026-05-13

**Authors:** Louise Rigny, Iona Biggart, Kimberley Zakka, Jingteng Li, Alexander Capstick, Sebin Sabu, Pavithra Rajendran, Shankar Sridharan, Sumera Farooq, Andrew Taylor, John Booth, Shiren Patel, Padmanabhan Ramnarayan, Neil Sebire, Cho Ng, Payam Barnaghi

**Affiliations:** 1 Department of Brain Sciences, Imperial College, London, UK; 2 Innovation and Virtual Environments, NIHR Great Ormond Street Hospital Biomedical Research Centre and GOSH Data Research, London, UK; 3 Care Research and Technology Centre, UK Dementia Research Institute, London, UK; 4 Department of Pediatrics, MedStar Georgetown University Hospital, Washington, DC USA; 5 Institute of Cardiovascular Science, University College London, London, UK; 6 Department of Surgery and Cancer, Imperial College, London, UK; 7 Imperial College Healthcare NHS Trust, St Mary’s Hospital, London, UK; 8 Department of Population, Policy and Practice, Institute of Child Health, University College London,, London, UK

**Keywords:** Paediatric research, Outcomes research, Risk factors, Translational research

## Abstract

Paediatric cardiology presents challenges due to the rarity and complexity of conditions like congenital heart disease. Using retrospective electronic healthcare records from 1,522 Great Ormond Street Hospital cases, we benchmark machine learning models to predict length of stay and retrieve similar patient cases. BioClinical-BERT is used for embedding-based retrieval, while a Random Forest model achieves the best length of stay prediction accuracy (0.88  ± 0.02), outperforming clinicians. The Random Forest model shows mean precision and sensitivity of 0.77  ± 0.03 and 0.76  ± 0.04 during NHS silent deployment (1,052 admissions). K-means identifies three clinically distinct subgroups. Cosine similarity retrieval reveals diagnosis-driven top matches, while complications dominate broader sets. In a 25-case intensive care unit pilot, clinician-rated utility improves from 4.23  ± 2.42 to 4.41  ± 2.16 (scale 0-10). Our models surpass clinician performance in length of stay prediction and show promise for case retrieval, supporting data-driven decision-making in paediatric cardiology and beyond.

## Abstract (web summary)

Paediatric heart conditions are rare and complex, complicating treatment planning. Here, the authors show that machine learning models can predict hospital stays and retrieve comparable cases, providing clinically useful insights for decision-support.

## Introduction

Children are significantly under-represented in clinical research worldwide^1,2^. Despite accounting for 21% of the UK population, only 3–4% of the healthcare research budget is designated to paediatrics^3^. Globally, a comparably limited proportion of the research resources is allocated to paediatric medicine^1^. This limits our understanding of paediatric diseases, particularly in complex fields such as cardiology^4^. Although paediatric cardiology has seen advances in the treatment of congenital heart diseases (CHDs) and surgical techniques, managing these conditions remains challenging. CHD and other paediatric heart diseases are rare and highly variable, making it difficult to study and predict outcomes. As a result, the rate of unexpected complications in paediatric cardiology is higher than in several other fields^5,6^.

The evolution of Artificial Intelligence (AI) in clinical environments offers a promising path to address these challenges by assisting clinical workflows, analysing heterogenous healthcare data and improving decision-making. AI tools are increasingly being applied to data from electronic health records (EHRs)—digital records that contain patients’ medical histories, diagnoses, treatment plans, and other clinical information over time. Deep learning (DL) models often perform well in automatically extracting features or variables from such complex, high-dimensional information, unlike other methods that require predefined features and thus prior knowledge for predictions^7,8^. These extracted features can then be used in downstream algorithms, including clustering and prediction models. Consequently, EHRs present an enriched training ground for DL methods due to their size, diversity, and variability, allowing for analysis of heterogeneous time-course medical data. The EHR data analysis can help identify patterns, trends, and potential risks that may not be immediately apparent to clinicians. With the widespread adoption of EHR systems, implemented in hospitals around the world^9,10^, the potential for DL applications in this domain is substantial. By using EHR data, clinical decision support (CDS) tools can improve diagnostic accuracy, personalise treatment plans, and optimise resource allocation. One outcome that has been frequently investigated in this context is LoS, which provides insight into both disease severity and hospital resource management. For example, Daghistani et al. developed a classification model to predict LoS in the cardiology unit, categorising stays as short (<3 days), intermediate (3–5 days), or long (>5 days), with the aim of addressing hospital bed capacity challenges^11^.

One promising approach within these tools is patient similarity analysis, which supports clinical decision-making by drawing on insights from patients with similar characteristics. This approach enables the development of AI and ML models that can provide clinical consultation support by presenting similar cases from historical data and predicting patient trajectories and outcomes. By embedding existing medical knowledge and expertise into EHRs and patient trajectories from highly specialised hospitals and centres, these models can extend useful insights to broader clinical settings, particularly in low-resource settings.

There are several existing methods in adult medicine that focus on patient similarity analysis. For example, Jia et al. proposed a non-deep learning model that predicted diagnoses by retrieving patients with similar diagnosis sets, while using dissimilar patients to filter out unlikely diagnoses^12^. Sun et al. demonstrated a dynamic patient similarity analysis model based on a long short-term memory (LSTM) network for diagnostic prediction and medication recommendation (Jaccard similarity: 0.4070; F1: 0.5672; sensitivity: 0.7832)^13^. Gupta et al. employed a supervised deep similarity learning approach based on a CNN architecture that learns patient representations and finds the relationship between patients using pairwise similarity^14^. Additionally, Oei et al. retrieved similar patients with diabetes mellitus by grouping patient profiles with clinical guidelines. Here, there was substantial agreement between their model and physicians (*κ* = 0.746)^15^. A study by Li et al. developed CHDmap, a patient similarity network to predict outcomes after CHD surgery, achieving competitive performance to clinicians^16^. However, the use of patient similarity retrieval methods and other decision support tools remains relatively limited, particularly in paediatric populations. Current tools in adult populations also face methodological limitations: (i) The concept of similarity is broad and multifaceted; (ii) current approaches often rely on simplistic criteria, such as the presence or absence of specific diseases, without considering the subtleties of individual patient histories and co-morbidities; (iii) common methods, such as neighbourhood-based algorithms, are limited in handling complex, multidimensional data; (iv) evaluation metrics largely rely on the Jaccard coefficient. In this context, the Jaccard coefficient quantifies the similarity between different fragments of EHR data. However, it does not account for the hierarchical relationships inherent in clinical data, such as those found in ICD coding systems^17^. Furthermore, existing patient similarity methods have often prioritised overall similarity in clinical profiles rather than specifically targeting similarities in clinical outcomes. As a result, important nuances in patient outcomes may be overlooked, potentially leading to sub-optimal recommendations and misclassification in both the training and inference phases.

Despite their potential for clinical translation, many ML models in clinical medicine are difficult to generalise beyond their development environments. The challenge lies in insufficient clinical evaluation, lack of real-world validation, and difficulty adapting to the dynamic nature of healthcare settings^18^. Several studies, such as those by Cabitza et al. and Wiens et al. emphasise that ML models such as large language models (LLMs) lack the deep contextual understanding and judgement that clinicians bring to patient care^19^. ML models often lack external validation and are not tested in real-world clinical settings. This leads to a discrepancy between their reported theoretical potential and practical utility^20^. Recent insights by Hager et al. underscore this issue, highlighting that while LLMs show promise, they currently fall short of autonomous clinical decision-making and perform less accurately than human clinicians in patient diagnosis^21^. This gap in validation contributes to a broader issue where tools may perform well under controlled conditions but lack reliability in real-world applications. Furthermore, many healthcare facilities lack the infrastructure for GPU-intensive tasks, which can inhibit model testing and integration in practice. In a recent study, Jia et al. demonstrated that insufficient computational resources (storage infrastructure, hardware, computing power) are challenged by the exponential growth in resources demanded by ML models in healthcare settings^22^.

**Fig. 1:**
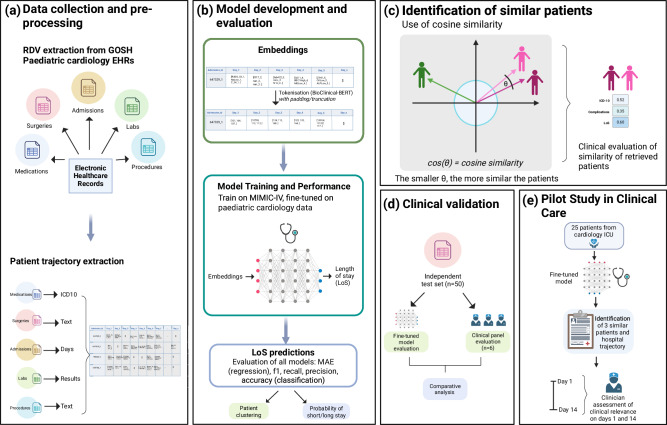
Study overview. The study uses clinical data from MIMIC-IV and GOSH cardiology electronic healthcare records, including admission data, diagnoses, medications, laboratory results, and procedures (**a**). Patient trajectories are extracted and embedded for input into a BioClinical-BERT model, initially trained on MIMIC-IV and fine-tuned with GOSH data. The model predicts LoS using one day of hospital data, predicting short/long stays (**b**) and identifying similar patients via a vector similarity measure based on extracted embedding vectors. The similarity measure identifies similar patients by calculating the cosine of the angle between the embedding vectors of two patients. The smaller the angle, the larger the similarity (**c**). A clinical panel (*n* = 6) reviewed an independent test set (*n* = 50) for comparison (**d**). A pilot trial assessed the fine-tuned model’s utility by presenting three similar patients to clinicians at the admission stage consultation from cardiology ICU wards at GOSH. Clinicians evaluate the relevance of these similar patients on the day of admission and again 2 weeks later (**e**). Created in BioRender. Rigny, L. (2026) https://BioRender.com/gtbyv2zLoS length of stay, EHR electronic healthcare records, MAE mean absolute error, RDV raw data version.

In light of these challenges, this study presents a clinically validated ML approach that uses EHR data to improve decision support for clinicians in paediatric cardiology. There remains an unmet need for research on patient similarity in paediatrics. Moreover, the heterogeneity of rare and complex diseases introduces an additional layer of difficulty for developing such models. Addressing this gap, we derive patient representations from EHR data to identify patients that are most similar in terms of complications, co-morbidities, and unplanned procedures. We then trained and tested a model to predict the LoS for paediatric cardiology patients and compared the predictions to those made by a panel of clinicians, serving as a use case to demonstrate the benefits of implementing a clinical ML model in these settings. We also conducted a 6-month silent mode deployment in the UK National Health Service (NHS) setting at the Great Ormond Street Hospital (GOSH) for Children. We then extended the model to identify and validate similar patients by computing the cosine similarity of trained embeddings - numerical representations of patient data that capture meaningful patterns learned by the model - via clustering analysis and *@n* retrieval analysis. Since the model is trained on Length of Stay (LoS), the resulting embeddings capture underlying patterns associated with unplanned events and complications, enabling improved clinically meaningful similar case retrieval. This similar case presentation and analysis offer clinicians insights into historical decisions, events, and outcomes from identified analogous cases to support their decision-making processes. A schematic overview of the framework is provided in Fig. 1. The model runs on a Central Processing Unit (CPU), making it suitable for low-compute environments and facilitating its implementation in resource-limited clinical environments.

## Results

### Comparative evaluation of machine learning models

To determine the most effective ML model for classifying short and long hospital stays, we evaluated a range of approaches, including LLM-based architectures and other methods. In evaluating LoS predictions using 5-fold cross-validation, all models were tested on the same separate test set to ensure consistent performance comparisons (MIMIC-IV; *n* = 13755, GOSH; *n* = 302). For short stays, the Random Forest model achieved a sensitivity of 0.79 ± 0.08, specificity of 0.91 ± 0.03, precision of 0.80 ± 0.04, and an F1 score of 0.79 ± 0.08. For long stays, it reached 0.90 ± 0.03 in precision, 0.91 ± 0.03 in sensitivity, specificity of 0.79 ± 0.08, and an F1 score of 0.91 ± 0.02. The overall accuracy was 0.88 ± 0.02. In contrast, the Decision Tree model showed lower performance, with an overall accuracy of 0.60 ± 0.15 across short and long stays (Table 1). The performance results for models trained on MIMIC-IV only are provided in Supplementary Table S1. A bias-and-disparity analysis shows model performance across demographics: Random Forest metrics by ethnicity and sex appear in Supplementary Tables S2 and S3, while BioClinical-BERT metrics for the same subgroups are in Supplementary Tables S4 and S5. The confusion matrices for the Random Forest and BioClinical-BERT models are presented in Supplementary Table S6. Further stratification of both models was performed to enhance clinical applicability and confidence in the results, which are shown in Supplementary Table S7. Supplementary Fig. 1 presents the Random Forest explainability analysis, highlighting the 20 most positive and negative feature contributions to the long-stay class.

**Table 1:** Performance metrics for models trained and/or evaluated on GOSH, MIMIC-GOSH, or traditional ML approaches

LoS	Short stay				Long stay				
	Sensitivity	Specificity	Precision	F1 score	Sensitivity	Specificity	Precision	F1 score	Accuracy
Deep learning									
BioClinical BERT (FT-GOSH)	0.81 ± 0.11	0.90 ± 0.06	0.74 ± 0.16	0.77 ± 0.13	0.90 ± 0.06	0.81 ± 0.11	0.93 ± 0.03	0.91 ± 0.04	0.87 ± 0.06
BioClinical BERT (FT-MIMIC-GOSH)	**0.77** ± **0.14**	**0.94** ± **0.02**	**0.82** ± **0.05**	**0.79** ± **0.09**	**0.94** ± **0.02**	**0.77** ± **0.14**	**0.92** ± **0.93**	**0.93** ± **0.02**	**0.89** ± **0.04**
ROBERTA (FT-GOSH)	0.72 ± 0.14	0.93 ± 0.04	0.78 ± 0.14	0.74 ± 0.13	0.93 ± 0.04	0.72 ± 0.14	0.90 ± 0.04	0.91 ± 0.03	0.87 ± 0.05
ROBERTA (FT-MIMIC-GOSH)	0.80 ± 0.04	0.85 ± 0.06	0.67 ± 0.09	0.72 ± 0.10	0.85 ± 0.06	0.80 ± 0.04	0.93 ± 0.04	0.89 ± 0.03	0.84 ± 0.04
Other DL									
LSTM GOSH	0.68 ± 0.06	0.60 ± 0.06	0.39 ± 0.04	0.49 ± 0.04	0.60 ± 0.06	0.68 ± 0.06	0.83 ± 0.05	0.70 ± 0.05	0.62 ± 0.04
Other ML									
Decision Tree	0.62 ± 0.23	0.57 ± 0.20	0.53 ± 0.22	0.54 ± 0.18	0.57 ± 0.20	0.62 ± 0.23	0.70 ± 0.27	0.59 ± 0.18	0.60 ± 0.15
Random forest	**0.79** ± **0.08**	**0.91** ± **0.03**	**0.80** ± **0.04**	**0.79** ± **0.08**	**0.91** ± **0.03**	**0.79** ± **0.08**	**0.90** ± **0.03**	**0.91** ± **0.02**	**0.88** ± **0.02**
Gradient boosting	0.76 ± 0.08	0.90 ± 0.02	0.76 ± 0.05	0.76 ± 0.06	0.90 ± 0.02	0.90 ± 0.02	0.90 ± 0.03	0.90 ± 0.03	0.86 ± 0.04

Accuracy represents the overall accuracy across both short and long stay. Best results in each category are highlighted in bold.

*FT-{x}* Fine-tuned with dataset from x, *DL* deep learning, LoS length of stay, ML machine learning.

### Clinical panel vs. model

**Fig. 2:**
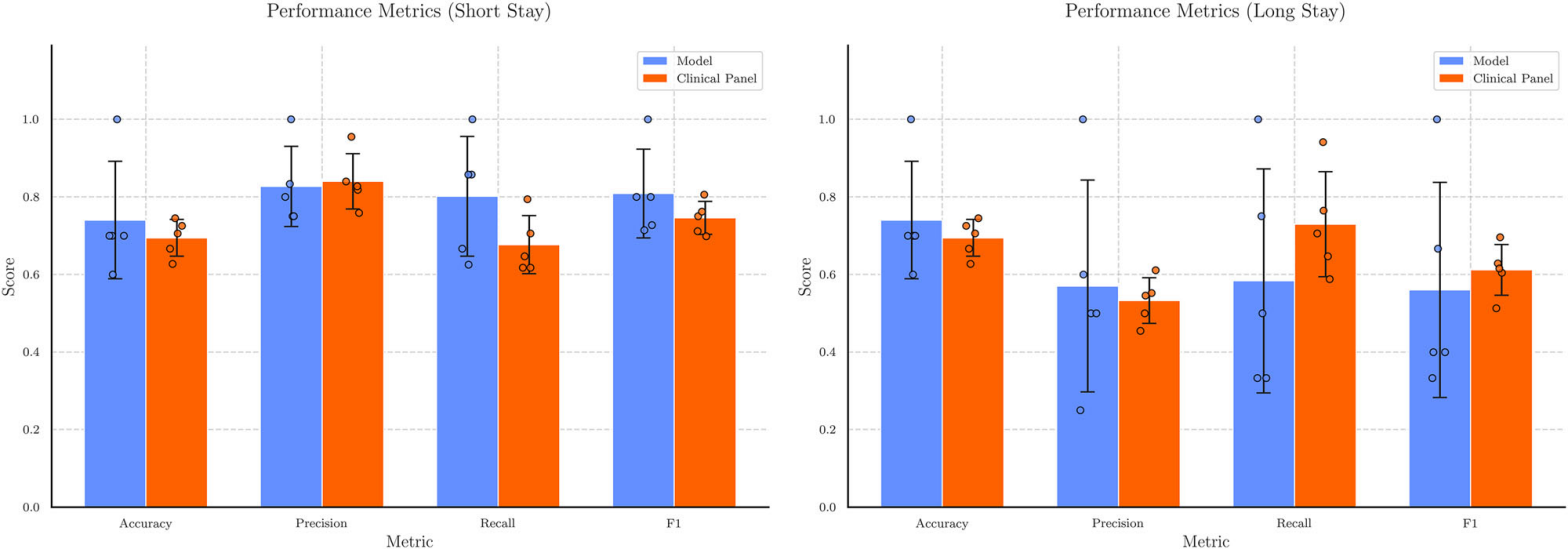
Performance comparison between clinical panel and model. Performance metrics (accuracy, precision, sensitivity, F1) of the BioClinical-BERT model, fine-tuned on the MIMIC dataset and subsequently on the GOSH dataset, for a) short-stay (1–3 days) and b) long-stay (4+ days) patient classifications. Error bars indicate the mean ± standard deviation across *k* = 5 folds of the model’s test set predictions (*n* = 50 patients) and from clinicians (*n* = 6 clinicians) in the clinical panel predictions. The patient cohort presented to the model and to the clinicians was identical. Overlaid points represent individual patient-level observations. Source data are provided as a Source data file.

To assess the clinical utility of the MIMIC-IV trained and fine-tuned cardiology classification GOSH model, a panel of 6 specialist and non-specialist clinicians in the domain reviewed a set of test patients (*n* = 50) to evaluate and predict the outcomes of their stay length. This was compared with the performance of the trained classification model on the same test set. The model outperformed the clinical panel on overall accuracy, short stay recall, short stay F1 score, and long stay precision (Fig. 2). The primary diagnoses and procedures of the patients misclassified by the clinical panel, but correctly identified by the model can be seen in Table 2.

**Table 2:** Overview of primary diagnoses and procedures of patient cases correctly classified by model but misclassified by 50%, 75% and 100% of clinical panel members (*n* = 6)

% of Clinicians who misclassified patient	Primary diagnoses (*n*)	Procedure (*n*)
50%	Ventricular septal defect (1) Ebstein’s anomaly (1) Marfan syndrome (1)	Repair of defect of interventricular septum (1) Percutaneous transluminal electroanatomic mapping (1) Other specified microtherapuetic endoscopic operations on larynx (1) Primary repair of defect of interatrial septum (1)
100%	Cardiac arrest, unspecified; other restrictive cardiomyopathy (1)	Exploratory median sternotomy (1) unspecified: diagnostic transluminal operations on heart (1)

### Silent deployment performance

**Fig. 3:**
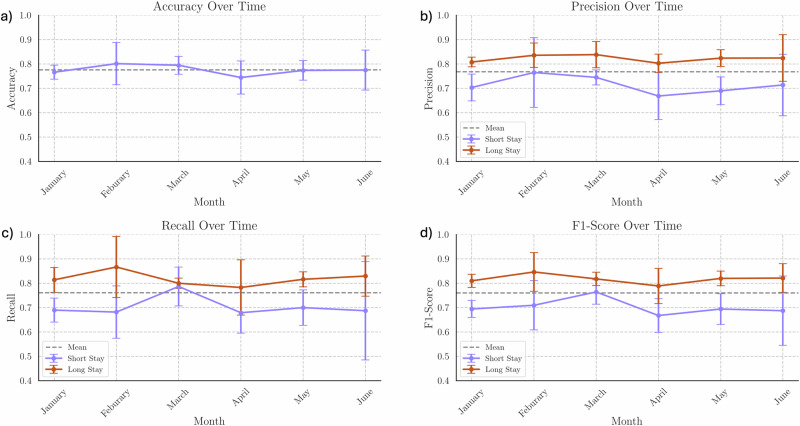
Silent deployment performance (Random Forest). Panels show performance metrics (**a**) accuracy, **b** precision, **c** sensitivity, and **d** F1-score) aggregated by 1-month intervals across 01/01/24–31/06/24. Monthly sample sizes were: January (*n* = 218), February (*n *= 161), March (*n *= 166), April (*n *= 184), May (*n *= 181), and June (*n *= 142). Error bars represent mean accuracy ± standard deviation. Grey dotted lines represent the mean value across the 6-month period. Source data are provided as a Source data file.

The Random Forest model to predict the LoS of the patient (short stay, long stay) was deployed within GOSH, processing real-time admission data over a period of 6 months. The results were consistent with the test set evaluations. During the silent mode deployment, the model achieved a mean accuracy of 0.776 (±0.021). For Short Stays metrics, mean precision was 0.714 (±0.036), sensitivity was 0.704 (± 0.041), and F1-score was 0.703 (±0.033). For long stays, the metrics showed a mean precision of 0.822 (±0.014), sensitivity of 0.818 (±0.029), and F1-score of 0.817 (±0.019). The average revealed a precision of 0.768 (±0.025), sensitivity of 0.761 (±0.035), and F1-score of 0.760 (±0.026). Figure 3 illustrates the model’s performance between 01/01/24 and 31/06/24. Silent deployment results using BioClinical-BERT are shown in Supplementary Fig. SF2.

### Patient Similarity Validation

**Fig. 4:**
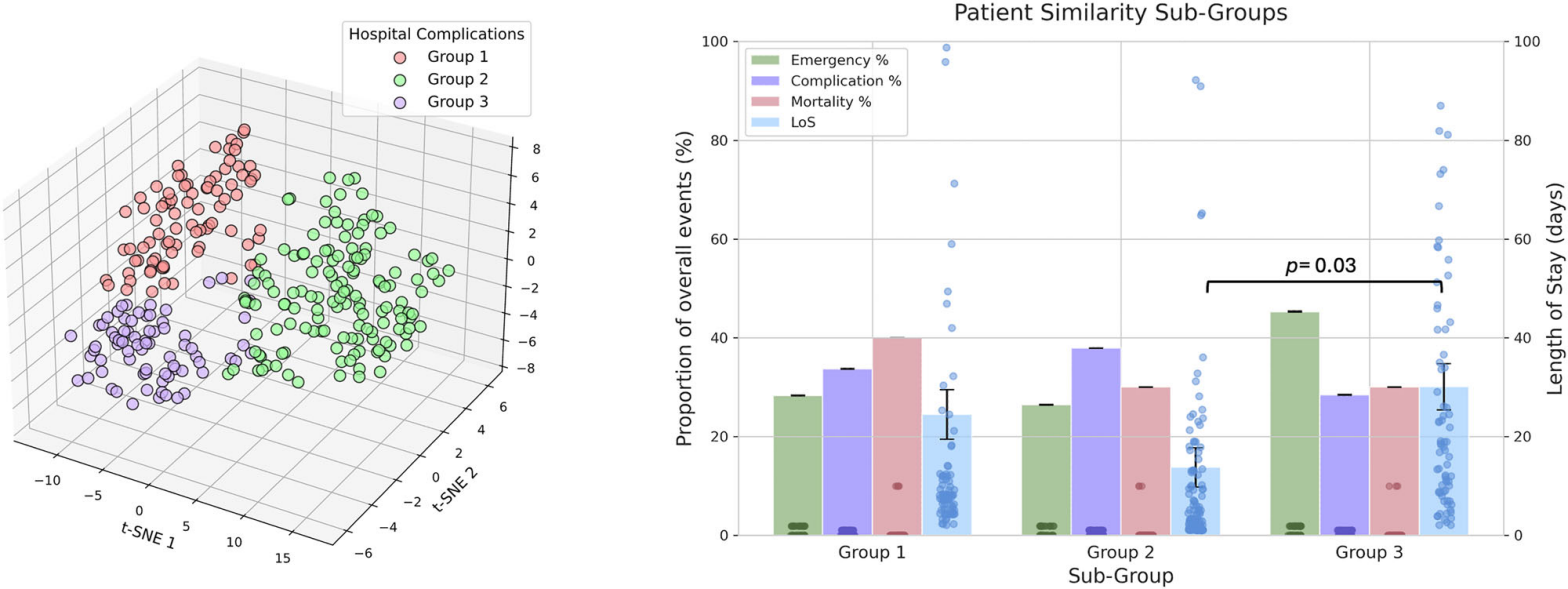
Patient sub-groups and associated hospital outcomes. Visualisation of **a** three-dimensional t-SNE representation of GOSH patient subgroups based on K-Means clustering with cosine similarity (*k* = 3), with cluster sizes: cluster 1 (*n *= 84), cluster 2 (*n *= 139) and cluster 3 (*n *= 74); **b** statistical analysis of clinical outcomes across the identified sub-groups, including the proportion of total mortality, complications and emergency events attributed to each cluster (summing to 100% across the three clusters), as well as average length of stay (LoS). Horizontal bars indicate statistically significant differences between groups (two-sided independent t-tests for LoS). Error bars represent the standard error of the mean. P-values were adjusted for multiple comparisons using Bonferroni-corrected post hoc comparisons. Overlaid points represent individual patient-level observations. Source data are provided as a Source data file.

Table 3 summarises the representative diagnoses, procedures, and exact similarity scores for each patient retrieved @ 1, 3, 5, and 10. Figure 4 presents an analysis of patient similarity sub-groups based on clinical data and their associated hospital outcomes (mean LoS, rate of complications, emergency events and mortality). Panel a) displays a 3D visualisation of the grouping of paediatric cardiology patients with GOSH into three sub-groups derived from dimensionality reduction techniques, illustrating natural separations based on length of stay, complication and emergency event rates. Panel (b) provides a quantitative comparison of hospital outcomes across these sub-groups. The bar plot shows the rate of emergency admissions, the rate of complication occurrences, and the average length of hospital stay (LoS, blue bars; right *y*-axis). A one-way ANOVA revealed a significant effect of group on mean LoS (*F*(294) = 3.56, *p* = 0.03, *η*^2^ = 0.024). Bonferroni-corrected post-hoc comparisons indicated that group 2 differed significantly from group 3 (*p* = 0.03, Hedges’ *g* = −0.37, 95% CI [−0.65, −0.08]). No significant difference was observed between groups 1 and 2 (*p* = 0.28). Hedges’ *g* = − 0.23, 95% CI [−0.50, 0.04]) or between groups 1 and 3 (*p* = 1.00, Hedges’ *g* = − 0.13, 95% CI [−0.18, 0.44]). A summary of representative diagnoses and procedures for each group is provided in Table 4.

**Table 3:** Retrieval patient similarity scores: analysis across different retrieval set sizes (@1, @3, @5, @10)

	GOSH—Retrieval similarity			
Metric	@1	@3	@5	@10
Cosine similarity	0.724 ± 0.042	0.723 ± 0.04	0.722 ± 0.042	0.72 ± 0.042
Hierarchical similarity mapping (Jia et al.)^64^	0.65 ± 0.12	0.61 ± 0.09	0.59 ± 0.09	0.56 ± 0.09
Primary diagnosis	0.23 ± 0.35	0.25 ± 0.60	0.31 ± 0.31	0.33 ± 0.30
All diagnoses	0.47 ± 0.35	0.51 ± 0.16	0.53 ± 0.14	0.65 ± 0.60
Procedures	0.87 ± 0.60	0.85 ± 0.60	0.84 ± 0.58	0.65 ± 0.61
Proportion complications	0.52 ± 0.31	0.50 ± 0.21	0.48 ± 0.16	0.47 ± 0.13
Proportion LoS category	0.48 ± 0.50	0.50 ± 0.41	0.55 ± 0.31	0.59 ± 0.27
Overall (*S*_overall_)	0.51 ± 0.42	0.52 ± 0.40	0.54 ± 0.30	0.54 ± 0.38

Primary and all diagnoses are based on ICD-10 classification. Procedures are based on the Open Contracting Data Standard (OCDS)-10 coding system. Complication and LoS similarity are measured as the proportion of query-retrieved patient pairs with a binary match. Error values represent standard deviation.

**Table 4:** Patient profiling of clusters

Cluster	Cluster size (*n*)	Top 5 primary diagnoses (% diagnosis)	Top 5 procedures (% procedure)	Stability score (95% CI)	Cluster description
1	84	(1) Other physical therapy (40.82%) (2) Dietary counselling and surveillance (34.84%) (3) Acute pain (45.07%) (4) Atrial septal defect (29.25%) (5) Occupational therapy and vocational rehabilitation NEC (37.04%)	(1) Posterior attachment of correctional instrument to spine (100%) (2) Left ventricle to aorta tunnel with right ventricle to pulmonary trunk direct anastomosis (100%) (3) Circumcision (100%) (4) Plastic repair of aorta (100%) (5) Repair of cor triatriatum (100%)	1.0 (1.0, 1.0)	Higher rate of mortality compared to Clusters 2 and 3
2	139	(1) Dietary counselling and surveillance (27.1%) (2) Other physical therapy (26.53%) (3) Atrial septal defect (32.08%) (4) Cardiomegaly (35.71%) (5) Acute pain (40.85%)	(1) Diagnostic transluminal operations on heart (75.0%) (2) Suture of chest wall (33.33%) (3) Unspecified repair of defect of interventricular septum (33.33%) (4) Other specified microtherapeutic endoscopic operations on larynx (50.0%) (5) Creation of anastomosis to pulmonary artery from vena cava (50.0%)	1.0 (1.0, 1.0)	Higher rates of complications and shorter LoS compared to Cluster 1 and 3.
3	74	(1) Dietary counselling and surveillance (38.06%) (2) Other physical therapy (32.65%) (3) Atrial septal defect (38.68%) (4) Occupational therapy and vocational rehabilitation NEC (31.38%) (5) Cardiomegaly (29.76%)	(1) Repositioning of transposed great arteries (75.0%) (2) Suture of chest wall (50.0%) (3) Unspecified repair of tetralogy of fallot (50.0%) (4) Plastic repair of aorta and end to end anastomosis of aorta (100%) (5) Application of band to pulmonary artery (50.0%)	1.0 (1.0, 1.0)	Higher emergency rate. Significantly higher LoS than Cluster 2

The percentage of procedures or diagnoses represents their proportion relative to total occurrences across all clusters. The stability score (0–1) quantifies the consistency of patient cluster assignments across multiple K-Means runs, with higher scores indicating greater stability. Additional profiling, including sex, age, ethnicity, mean LoS, complication rates, emergency events, and mortality, is provided in Supplementary Table 11.

*CI* confidence interval, *LoS* length of stay.

### Pilot study in clinical care

For the pilot study, patient embeddings from the fine-tuned BioClinical-BERT model, along with cosine similarity, were used to retrieve similar cases during clinical consultations. This model was selected because it demonstrated the best performance among deep learning-based embedding models for predicting prolonged LoS, as shown in Table 1. Clinician feedback on the utility of the approach was gathered on a 0–10 scale across six trial days. A sample of query patients and their corresponding predictions is shown in Supplementary Fig. SF3. It is important to note that, in this case, the aim is to achieve optimal information gain. In other words, the model should provide useful information without being redundant. If the model continually offers information that clinicians already know (very high score) or provides cases that are not useful (very low score), it will lack sufficient utility in real-world settings. An effective model should strike a balance between these two extremes. The utility of the model in real-world clinical settings depends on its ability to enhance the decisions made by clinicians and to highlight cases and potential complications that may not be immediately considered instantly^23^. Existing work on measuring the agreement between two independent raters (in this case, the clinician and the model’s rating of similarity/usefulness) indicates that moderate agreement will yield an optimal model^24^.

The mean utility scores for each trial day, along with feedback collected 2 weeks post-admission, are summarised in Table 5. The results indicate that clinician utility scores for the approach varied across the trial days, with an average score of 4.23 ± 2.42. Two weeks after admission, the mean score increased slightly to 4.41 ± 2.16. The mean scores indicate that the model offers insights that extend beyond those a clinician would have considered at the time of consultation without presenting a lot of redundant or irrelevant information. A sample of a query patient, along with a corresponding prediction for a similar patient and their hospital trajectory, is shown in Supplementary Fig. SF3.

**Table 5:** Clinician rankings of the utility of retrieved patients in clinical care

Scoring date	Utility scoring, mean ± SD	Median	Feedback (%)
Day of admission	4.23 ± 2.42	4.0	Clinical relevance and applicability (52.0%) Insights about adverse events (14.0%) Insights about LoS (8.0%)
2 weeks post admission	4.41 ± 2.16	4.0	Clinical relevance and applicability (63.70%) Insights about adverse events (23.11%) Insights about LoS (33.52%)

Rankings represent the mean utility score (±standard deviation) across retrieved patients for each day. Feedback is reported as the percentage of cases that demonstrated insight within each category. This score indicates higher utility of the model without mostly confirming what clinicians already knew or providing information that clinicians do not find useful.

*SD* standard deviation.

## Discussion

We present a clinically validated and data-driven approach using EHR data to enhance decision support in paediatric cardiology. Our primary objective was to use ML models to derive patient representations from EHR data, identifying similar profiles based on complications, comorbidities and unplanned procedures.

A range of ML models were trained on MIMIC-IV ICU data and further fine-tuned on the GOSH cardiology dataset to predict LoS categories; short-stay (1–3 days) and long-stay (4+ days) using the first day of hospital admission data. The Random Forest Model performed best, demonstrating an overall accuracy of 88% across both stay categories. The BioClinical-BERT model was the best-performing deep learning model, which was used for generating embeddings to identify similar patients. Compared to a panel of 6 clinicians, consisting of domain specialists and non-specialists, the model outperformed on the majority of metrics. This is in agreement with previous work showing that ML models can outperform clinicians and potentially support clinical decision-making^25–28^. For example, a study by Rajkomar and colleagues demonstrated that ML models could outperform clinicians in tasks such as early detection of sepsis^29^. Traditional regression analyses are frequently used for LoS prediction^30,31^, yet our study found that a regression model implemented using a Random Forest model was less accurate than the classification approach (see Supplementary Fig. SF4). This may be attributed to the nature of the training dataset, which was derived from paediatric patients at GOSH, which is a centre for rare and complex diseases. This meant that identifying clear patterns for regression prediction is inherently challenging. We also focused on the utility and real-world application of the model. Instead of giving a predicted LoS as a number, we provide a categorical view (short and long stay), which can help with bed management and treatment planning. The binary classification approach yielded promising results, achieving an overall accuracy of 88% for the Random Forest Classifier and 89% for the BioClinical-BERT model. This approach is useful in clinical settings as bed management systems prioritise identifying patients likely to stay beyond a threshold, enabling better resource planning^32,33^. Systematic reviews highlight the role of dedicated discharge planning teams in following long-term patients to address the causes of extended stays and optimising overall bed use^34^. Given the challenges of modelling rare paediatric disease data, future work may consider alternative approaches like temporal convolutional networks, which have been shown to capture spatio-temporal patterns effectively, may further enhance performance^35,36^.

The Random Forest model was silently deployed within the DRE at GOSH, processing real-time admission data over 6 months (01/01/24–31/06/24). It demonstrated consistent performance, maintaining a high overall accuracy of 0.766 ± 0.021. While precision (0.768 ± 0.025) and sensitivity (0.761 ± 0.035) were balanced, they were slightly lower for short stays. This discrepancy may reflect a common challenge noted in previous research, where predicting shorter stays is inherently more complex due to variability, particularly in paediatric settings^31,32,37–40^. Nevertheless, the model’s overall performance remains robust, demonstrating strong generalisability and clinical applicability even in the presence of such variability. Our study’s silent deployment aligns with guidelines for evaluating ML models before clinical integration. Tonekaboni et al. introduced a silent trial protocol to test real-time models in ICU settings, emphasising the need to address issues such as data set drift, bias and feasibility before models influence patient care^41^. Similarly, another study conducting a silent trial of an AI model designed to predict obstructive hydro-nephrosis in infants revealed significant issues when tested on prospective data, with performance dropping from an AUC of 0.90–0.50^42^. These works underscore the importance of silent deployments as a bridge between algorithm development and clinical adoption. However, such applications remain relatively limited in paediatric settings, particularly in the context of rare and complex diseases. The unique challenges posed by these conditions, such as smaller datasets and variability in disease presentation, make silent deployment in this domain necessary and under-explored. Our work contributes to this limited research on deploying AI models in specialised paediatric settings.

The patient similarity clustering analysis revealed three groups based on LoS, number of complications and emergency procedures. Group 3 had a significantly higher LoS compared to Group 2 (*p* = 0.03). In terms of emergency rates, Group 3 also exhibited higher rates than Group 2. These findings align with clinical expectations, as the procedures in each group reflect the observed differences in LoS and complications. Group 3, which had a higher LoS and emergency rate, included patients undergoing more complex and invasive surgical interventions, such as heart transplantation and Tetralogy of Fallot repair. These patients typically require longer hospital stays, ranging from 19 to 30 days, depending on factors such as age and the aetiology of organ failure (CHD versus cardiomyopathy)^43^. Group 2, which had a significantly lower LoS than Group 3, comprised patients characterised by predominantly lower-acuity diagnoses and less invasive procedures. Finally, Group 1 included patients with broader ICD diagnoses (e.g., ‘other physical therapy’) and complex procedures, including ‘repair of aorta’ and ‘repair of cor triatriatum’.

Risk modelling and stratification in cardiothoracic procedures, particularly those related to CHD remain challenging, largely due to institutional preferences, disease heterogeneity and surgeon-specific practices^44^. Our model demonstrates promise in navigating these complexities. By using ML-based CDS, healthcare teams can more effectively allocate resources and interventions to high-risk patients. These findings support previous work in other medical contexts, where ML models have successfully predicted adverse outcomes for inpatient populations based on early hospital data^45,46^. Additionally, the model is based on the BioClinical-BERT architecture, which has reduced computational requirements compared to larger transformer models. BioClinical-BERT has approximately ~110 million parameters (12 layers, 768 hidden size, 12 attention heads), making it smaller than general-domain models like BERT-Large, which has ~340 million parameters. This smaller size translates to faster inference times and lower hardware demands and is manageable to run on a CPU rather than complex models that require GPU resources. This manageable demand for computing resources makes the trained model particularly useful for resource-constrained environments in clinical and biomedical applications.

Our findings demonstrate that the learned patient embeddings effectively encode LoS at a high level, as evidenced by the significant LoS differences observed between clusters. However, within each cluster, retrieval rankings appear to be influenced by complications rather than LoS, with complication similarity decreasing as the number of retrieved patients increases. This suggests that while LoS is encoded at a broad level, it is not a primary factor in fine-grained patient similarity within clusters. Diagnosis strongly influences early retrieval (@1), while other clinical factors,including complications, play a more prominent role at larger retrieval sets (@3, @5, @10). Since complications were a main goal, these results indicate that the model successfully captures both cluster-level and retrieval-level complication similarity, ensuring that retrieved patients share meaningful clinical characteristics beyond LoS. A limitation of the current similarity approach is using cross-entropy loss for LoS categories (Short vs. Long Stay), which trains for decision boundaries but does not preserve intra-category similarity. Due to this, clusters are broadly separated by LoS, but retrieval rankings prioritise complications and diagnoses. To improve retrieval alignment with LoS, future work can incorporate contrastive loss functions (e.g., Triplet Loss, NT-Xent) to explicitly enforce LoS-based ordering within clusters, ensuring that retrieved patients share not just categorical LoS labels but also finer-grained LoS similarity. By refining these aspects, we can develop a more clinically meaningful patient retrieval framework, enhancing its utility for personalised treatment planning.

A pilot study in ICU wards assessed the practical utility of the fine-tuned BioClinical-BERT model for LoS prediction and patient similarity. Over 6 days, clinicians received predicted LoS and three similar patient cases for newly admitted patients, providing feedback on admission and 2 weeks later (rated 1–10). The average utility score was 4.23 ± 2.42 on admission and 4.41 ± 2.16 post-admission. The mean score is significant as it indicates that the model offers insights that extend beyond those a clinician would have considered at the time of consultation without presenting a lot of redundant or irrelevant information. A very high or a very low score would not have corresponded to a significant clinical utility. Clinicians found clinical relevance/applicability in 63.7% of cases, adverse event information in 23.11% and LoS insights in 33.5%. The study demonstrates the model’s potential clinical value, with clinicians finding it most useful for general relevance (63.7%) and showing stable utility over time. These results highlight its promise in supporting decision-making and suggest that further refinements in patient similarity retrieval and interpretability could enhance real-world applicability.

This study focused on cardiology wards to ensure a controlled evaluation within a high-risk patient cohort where accurate predictions are particularly helpful due to the inherent variability of its conditions. However, it did not account for transfers or other specialities. Future work could extend this approach to a broader range of specialities and a larger sample size. Additionally, the analysis used 2 years of post-pandemic data (2021–2023) to minimise potential confounding effects from COVID-19, such as shifts in admission patterns, resource availability and discharge practices^47–49^. Incorporating a longer longitudinal sample could further enhance model reliability.

Overall, this study provides a proof of concept within a paediatric cardiology unit, providing an AI-based tool to work in collaboration with clinical experts. Further work is needed to evaluate model performance across different medical specialities. Given the standardised data representations and diagnosis codes used, the proposed methodology and model architecture are transferable and scalable to other hospital settings. Moreover, while this analysis considered age, sex and ethnicity, future studies should further assess potential variability and bias across demographic groups.

## Methods

An overview of our data collection and pre-processing, model development, and examples of the clinical applications are presented in Fig. 1 and discussed in this section.

### Data Collection and Population

This study was conducted in collaboration with Imperial College London and Great Ormond Street Hospital for Children (GOSH). Data was extracted from GOSH’s EHR system and accessed via GOSH’s secure Digital Research Environment (DRE). All records were anonymised in accordance with GOSH’s approved data-use protocol. The study was registered with the GOSH R&D department. The study was approved by the Health Research Authority (HRA)/REC as secondary use of data (HRA/REC 21/LO/0646). It used non-identifiable data from the GOSH digital research environment. As this study used only anonymised, routinely collected machine data from NHS records, individual patient consent was not required under the terms of the ethical approval and in accordance with NHS research governance frameworks for anonymised data. No data from GOSH was shared outside the organisation, and all analyses were conducted within the GOSH DRE, ensuring compliance with data security and governance protocols. MIMIC-IV data is fully de-identified, and the Beth Israel Deaconess Medical Center (BIDMC) institutional Review Board has granted a waiver of informed consent and approved the sharing of the research resource.

Two datasets were used to test and train the model: (i) A de-identified paediatric cardiology dataset extracted from the GOSH EHR database, including patients admitted to cardiology, cardiothoracic surgery, or paediatric cardiology hospital services from June 2021 to June 2023. This dataset is referred to as the GOSH cohort in this paper and consists of data from 1522 individuals; (ii) MIMIC-IV, a publicly available dataset curated and shared by Bulgarelli et al. at the Massachusetts Institute of Technology (MIT)^50^. MIMIC contains de-identified data at the bedside for patients cared for in an intensive care unit (ICU) at the Beth Israel Deaconess Medical Center (BIDMC) between 2008 and 2019^50^.

The model was first trained on MIMIC-IV. Despite physiological differences between the adult and paediatric populations, we investigated whether the models pre-trained on MIMIC-IV could provide better performance once further trained on specialised paediatric EHR data. This pre-training step allows the model to learn general physiological patterns, relationships, and clinical trends across a wide range of patients before fine-tuning on the GOSH dataset^51,52^. Given the limited availability of rare disease data, this approach helps enhance model performance while mitigating over-fitting. The total MIMIC-IV and GOSH datasets used for this study include EHR data from 70,068 patients. Patient demographics for both datasets are summarised in Table 6, with demographic group categorisations detailed in Supplementary Table S8.

**Table 6:** Selected demographic and statistical information for pre-processed MIMIC-IV and GOSH datasets

	MIMIC-IV		GOSH	
	Train/Validation	Test	Train/Validation	Test
Patients	54,791	13,755	1220	302
Sex				
Male	31,051 (56.67%)	7699 (55.97%)	706 (57.86%)	172 (56.95%)
Female	23,740 (43.32%)	6056 (44.02%)	514 (42.13%)	130 (43.04%)
Age				
Min	18.00	18.00	1.3	1.3
Max	103.00	102.00	20.90	20.70
Mean ± SD	65.09 ± 16.44	65.12 ± 16.54	7.54 ± 5.72	7.22 ± 5.68
Race				
White	34,888 (63.67%)	8732 (63.48%)	560 (45.90%)	123 (40.73%)
Black	4866 (8.88%)	1194 (8.68%)	95 (7.79%)	33 (10.93%)
Asian	1504 (2.74%)	388 (2.82%)	233 (19.10%)	53 (17.55%)
Mixed	46 (0.08%)	7 (0.05%)	66 (5.41%)	21 (6.95%)
Other	6983 (12.74%)	1758 (12.78%)	160 (13.11%)	46 (15.23%)
N/A	6504 (11.87%)	1676 (12.18%)	106 (8.69%)	26 (8.61%)
Mortality	6058 (11.06%)	1581 (11.49%)	45 (3.69%)	10 (3.11%)
LoS				
Short stay	32,626 (59.55%)	8125 (59.07%)	364 (29.84%)	82 (27.14%)
Long stay	22,165 (40.45%)	5630 (40.93%)	856 (70.16%)	220 (72.85%)

*N/A* not available, *SD* standard deviation, *LoS* length of stay.

### Data pre-processing and timeline extraction

To train the models, we use the MIMIC-IV ICU dataset and a fully anonymised dataset from the GOSH paediatric cardiology, which includes demographics (age, sex, ethnicity), admission and discharge dates, procedures, administered medications, laboratory measurements and diagnoses. Diagnoses follow the International Classification of Diseases, 10th Edition (ICD-10) classification system^53^. Procedures follow the Office of Population Censuses and Surveys Classification of Interventions and Procedures, Version 4 (OPCS-4) system^54^. Each hospital stay was uniquely identified by an admission ID assigned to each individual stay. We filtered the dataset to include only in-patient cases, excluding day cases. The latter was due to the main objective of the study, which was to estimate the LoS and the patient trajectory in hospital by analysing previously encountered similar cases. Any medical events recorded outside the patient’s admission-to-discharge window were removed. For timeline extraction, each day of a patient’s hospital stay was mapped to a 24-h window relative to their admission and discharge dates. Whilst diagnoses (based on ICD-10 descriptions) were collected throughout the admission process for the MIMIC-IV dataset, this was not the case for the GOSH dataset. At GOSH, initial diagnosis codes were assumed to be collected prior to admission and were assigned on Day 1 for each admission ID. This reflects the specialised nature of GOSH, a hospital that receives referrals from national and international clinical centres. Given this referral process, patients often arrive with a preliminary diagnosis already established by their referring clinicians. The events from the day of admission were formatted into one long string of events and used as input data for model training. Only the data from the first day of each admission ID was used to predict the LoS. We consulted with the clinical teams and reviewed the existing literature^11,34,35,55^ to determine the most effective way to represent the LoS. In the final results, LoS labels were categorised as short-stay (1–3 days) and long-stay (4+ days). This LoS estimate can be updated daily for each individual based on information regarding the following day’s stay.

### Model comparison and rule-based approaches

To determine the most effective ML model for classifying short and long hospital stays, we evaluated a variety of approaches, including DL models with lower computational requirements, LLM architectures and other methods. A rule-based model was also developed to provide a comparative analysis of predictive performance between simpler rule-based and more complex LLM-based methods. For the rule-based method, we developed a Decision Tree classifier using encoded features that model clinical decision-making processes. Here, we encoded key features such as diagnosis, surgery, sex and age, which are integral to clinical workflows and have higher predictive power^32,56^. This model aimed to replicate the decision-making framework used by clinicians, offering a practical assessment. Secondly, a Random Forest classifier, a Gradient Boosting classifier and LLM-based methods were fitted on the same input data fed through a count vectoriser to represent the frequency of event occurrence, giving a direct technical comparison of performances.

To ensure the quality and comparability of the data, both training and test datasets were combined for consistent encoding. The encoded features were then standardised. To address the class imbalance within the training data, minority classes were upsampled. The models were trained on the pre-processed data and evaluated using 5-fold cross-validation with patient-level stratification to prevent leakage to ensure a reliable estimate of performance across multiple subsets of the data. Upsampling was applied only to the training folds, with validation sets left unchanged to ensure an unbiased evaluation.

### Model development

BioClinicalBERT was the best performing DL model for LoS prediction (Table 1) and was selected for further development. The performance results for models trained on MIMIC-IV only are reported in Supplementary Table S2. Beyond classification performance, its embeddings were used for downstream patient similarity analysis (see Patient Similarity Validation section), making it the most suitable choice for our study workflow. We trained two versions of BioClinicalBERT^57^ on MIMIC-IV before fine-tuning on 2 years (June 2021–June 2023) of GOSH paediatric cardiology data. Although BioClinicalBERT was initially trained on MIMIC datasets, we further trained it on MIMIC-IV for LoS classification to refine task-specific embeddings and align with the clinical language and structures relevant to LoS prediction. We then fine-tuned the pre-trained model on the GOSH dataset. Data was split into training, validation, and testing subsets (55:25:20) and tokenised using BioClinicalBERT. This model was chosen over BERT base-uncased as it was trained on clinical text to better capture medical terminology^58^. Key model parameters and specifications for BioClinical-BERT are provided in Supplementary Table S9.

We developed a binary classification BioClinicalBERT model to predict LoS categories using the first day of hospital admission data: short-stay (1–3 days) and long-stay (4+ days), using the model’s pre-existing classification head. Binary classification was chosen over regression for LoS prediction to improve clinical interpretability, mitigate data variability, and address the skewed LoS distribution (See Supplementary Fig. SF4). Since we use only the first day of hospital data, this approach allows for daily LoS prediction updates. Initial experiments have shown that regression-based models had higher variance and performed less reliably compared to classification models, which provided more stable and clinically useful predictions^33,59^. The results of a regression-based LoS analysis are reported in Supplementary Fig. SF4 and support these findings. Due to class imbalances, training data was resampled for equal class distribution. The BioClinical-BERT model was initialised with pre-trained weights and fine-tuned for 10 epochs using cross-entropy loss.

### Model evaluation

For technical validation, we used accuracy, precision, sensitivity, specificity and F1-score to assess the model’s predictive capability across all classes. A confusion matrix was used to evaluate classification errors and identify patterns of misclassification. A fairness and bias analysis was conducted for the best performing model by computing performance metrics across ethnicity categories (Asian, Black, Mixed, White) and sex (Female, Male) across 5-fold cross-validation (Supplementary Tables S2–5). All metrics were computed on an independent test set (MIMIC-IV; *n *= 13,755, GOSH; *n *= 302) and validated via 5-fold cross-validation to assess for generalisability. This evaluation was performed on the MIMIC-IV-trained model and its fine-tuned version using the GOSH dataset. Predictions were stratified into three groups according to their predicted class probabilities, with the stratification designed to maximise Youden’s *J* index.

1 $$J={{{\rm{Sensitivity}}}}+{{{\rm{Specificity}}}}-1$$

An explainability analysis for the Random-Forest model (top performing LoS model, see Table 1, showing the top 20 positive and negative feature contributions for long-stay class is provided in Supplementary Fig. 1 (SF1).

#### Patient similarity

The similarity measure uses the cosine of the angle between the two vectors in the high-dimensional embedding space, with values ranging from −1 (completely dissimilar) to +1 (completely similar), where a value of 0 indicates no similarity. Measuring the angular distance between high-dimensional patient vectors helps to identify patients with similar clinical profile embeddings^60^. Previous studies have shown effectiveness of this approach in identifying clinically meaningful patient relationships^61–63^. The cosine similarity between two patient embeddings, **v**_**1**_ and **v**_**2**_, derived from a high-dimensional vector representation, given by the following:

2 $$\,{{{\rm{Cosine\; Similarity}}}}\,({{{{\boldsymbol{v}}}}}_{1},{{{{\boldsymbol{v}}}}}_{2})=\frac{{{{{\boldsymbol{v}}}}}_{1}\cdot {{{{\boldsymbol{v}}}}}_{2}}{\parallel {{{{\boldsymbol{v}}}}}_{1}\parallel \parallel {{{{\boldsymbol{v}}}}}_{2}\parallel }$$

where

3 $${{{{\boldsymbol{v}}}}}_{1}\cdot {{{{\boldsymbol{v}}}}}_{2}={\sum }_{i=1}^{n}{v}_{1}^{i}{v}_{2}^{i}$$

is the dot product of the two vectors, and

4 $$\parallel {{{{\boldsymbol{v}}}}}_{1}\parallel=\sqrt{{\sum }_{i=1}^{n}{({v}_{1}^{i})}^{2}},\,\parallel {{{{\boldsymbol{v}}}}}_{2}\parallel=\sqrt{{\sum }_{i=1}^{n}{({v}_{2}^{i})}^{2}}$$

are the Euclidean norms (magnitudes) of the vectors **v**_**1**_ and **v**_**2**_, respectively, where ${v}_{1}^{i}$ and ${v}_{2}^{i}$ are the components of the vectors **v**_**1**_ and **v**_**2**_, and *n* is the number of dimensions (features) of the embeddings.

To validate patient similarity, two separate similarity approaches were implemented; an overall similarity score, *S*_overall_, and a hierarchical similarity mapping. *S*_overall_ was computed for each query patient and identified similar patients, as determined by embeddings with highest cosine similarity. The overall similarity is calculated as a weighted combination of primary and overall diagnosis similarity (*S*_Primarydiagnosis_), (*S*_Diagnoses_), procedural similarity, (${S}_{{{{\rm{Procedures}}}}}$) complication similarity (*S*_Complications_), and LoS similarity (*S*_LoS_). Diagnosis and procedural similarity were calculated by comparing ICD-10 codes and OPCS-4 codes, respectively, between the query patient and retrieved patients. The diagnosis codes were split into lists, allowing for pairwise comparisons. The length of the ICD-10 codes can vary from 3 to 7 characters, organised in a hierarchical manner^53^. For each pair of diagnosis codes, a score was computed based on the length of matching prefixes between the full ICD-10 codes (not individual characters), with the score incrementing based on the character position of each match. The total points obtained from all comparisons were normalised against the total possible points to derive a similarity score ranging from 0 to 1.

Complication similarity was computed by identifying the presence of specific diagnosis codes in both the query patient’s and the identified patient’s records. ICD-10 codes indicating post-procedural complications and disorders (‘T’ category codes), sepsis (A40, A41), and other major complications (e.g., respiratory (J95), cardiovascular (I97), and neurological (G97) were flagged. For each patient pair, if both had complications, the similarity score was set to 1. If neither had complications, the score was 0.5, and if only one had complications, the score was 0.

LoS similarity was calculated by categorising LoS values as Short Stay (≤3 days) or long stay (>3 days) and computed a similarity score (1 if categories matched, 0 otherwise), averaging across retrieved @n patients.

5 $${S}_{{{{\rm{Overall}}}}}=\frac{{w}_{1}\,{S}_{{{{\rm{Primary}}}}{{{\rm{diagnoses}}}}({{{\rm{ICD}}}}-10)}+{w}_{2}\,{S}_{{{{\rm{Diagnoses}}}}({{{\rm{ICD}}}}-10)}+{w}_{3}\,{S}_{{{{\rm{Procedures}}}}({{{\rm{OPCS}}}}-4)}+{w}_{4}\,{S}_{{{{\rm{Complications}}}}}+{w}_{5}\,{S}_{{{{\rm{LoS}}}}}}{{w}_{1}+{w}_{2}+{w}_{3}+{w}_{4}+{w}_{5}}$$

The hierarchical similarity mapping was inspired by the method outlined by Jia et al., where the similarity is quantified based on how close two patients’ diagnoses align^64^.

#### Unsupervised clustering analysis

In addition to retrieving similar cases to individual queries, we also conducted a group-level analysis. Using the embeddings generated by BioClinical-BERT for each patient’s day 1 EHR data, we identified patterns among patients with similar clinical characteristics. To achieve this, we applied unsupervised models to group patients into distinct clusters based on their clinical profiles (LoS, procedures, diagnoses, and age). First, patient embeddings were standardised using z-score normalisation. We then performed t-distributed Stochastic Neighbour Embedding (t-SNE) to reduce the dimensionality to three components. To determine the optimal clustering algorithm and number of clusters, we compared Agglomerative clustering, K-means with cosine similarity, and Spectral clustering. These were selected for their ability to capture hierarchical structure, cosine-based similarities in high-dimensional spaces, and graph-based relationships, respectively^65^. We evaluated clustering performance using the silhouette score, Davies–Bouldin (DB) index and Calinski–Harabasz (CH) index, computed across 100 bootstrap resamples to assess robustness. Based on these metrics, K-means with cosine similarity was selected with three clusters. The analysis results are available in Supplementary Fig. SF5 and Supplementary Table S10.

Clustering was repeated 10 times with different random seeds. Each patient’s final cluster was assigned based on their most frequent label across runs, and a stability score was computed as the proportion of runs in which the patient was consistently assigned to that cluster. A higher stability score signifies greater consistency in patient clustering across all iterations^66^. To profile clusters, we computed the top five primary diagnoses and procedures as a percentage of total occurrences, representing their proportion relative to all diagnoses or procedures across clusters (Table 4). As 5 patients from the test cohort lacked information regarding primary diagnoses, they were excluded from the clustering analysis. We also calculated mean age, LoS, complications and emergency and mortality rates, reporting 95% confidence intervals (CIs) using standard error-based estimation (Supplementary Table S11). Patient profiling was used to assess the significance of differences between features across clusters, and Analysis of Variance (ANOVA) and Tukey’s post hoc test were used to determine statistically significant differences between clusters. Fisher’s Exact test was used for categorical data (rate of complications, mortality, emergency events). Statistical results are reported in Supplementary Tables S12–16.

### Assessment of clinical utility

To assess the clinical utility of the cardiology-trained GOSH language model, a panel of 6 domain specialist and non-specialist paediatric clinicians reviewed a test set to evaluate and predict the LoS prediction results. This clinical validation involved assessing 50 randomly selected paediatric cardiology cases from GOSH, using the same test set to evaluate the performance of the trained model. The samples included those who were discharged back to their usual place of residence rather than to a different hospital ward. Clinicians were presented with data from the first day of hospital admission, including diagnoses (ICD-10), procedures, medications, surgeries, laboratory results, and demographic details (sex, race, and age). The clinicians predicted the LoS category (short-stay, long-stay) for each case based on standard clinical knowledge and case management guidelines. These clinician assessments were then compared to the model’s predictions to determine the model’s utility and its practical value in real-world clinical settings.

### Silent deployment within GOSH

We also evaluated the trained model’s performance with prospective data to assess the generalisation of the model to unseen data over a period of time using data from the hospital’s routine operations. The best-performing model to predict patient LoS was chosen for this purpose (Table 1). The model was integrated into the on-premise infrastructure to run on data from all admissions to cardiology, cardiothoracic surgery, and paediatric cardiology hospital services over a 6-month period, covering a diverse cohort of patients. Results were aggregated at 1-month intervals over a 6-month time frame (01/01/24–30/06/24) for 1052 admissions. The demographic data across the silent deployment period is provided in Supplementary Table S17. The model predictions were generated upon patient admission. Throughout the evaluation period, the model’s predictions were stored without clinician visibility, allowing for the collection of performance metrics (accuracy, precision, sensitivity, and F1-score) based on actual patient outcomes. This minimises potential biases and changes in clinical decision-making behaviour.

### Pilot study in clinical care

A pilot study was conducted across intensive care units (ICUs) at GOSH for patients with cardiac diagnoses to assess the utility of the developed method in identifying similar cases from historical data. The aim was to evaluate if presenting similar cases from historical data could provide new information and insights about patients at risk of future complications. Clinical data (demographics, diagnoses, and procedures) at the time of the first consultation for 25 patients over a week were submitted via a form (the form completion takes approximately 1 min per patient). Patient selection was flexible, requiring only anonymised diagnostic data for cases that were admitted during the pilot study period. Of the 25 patients, 60% were admitted to cardiac ICU (CICU), 8% to neonatal ICU (NICU), and 32% to paediatric ICU (PICU). The study used a fine-tuned BioClinical-BERT model to predict the length of hospital stays and provide information about potential complications based on retrieving similar cases from retrospective case data (see Section ‘Patient similarity validation’). Patient embeddings were generated from this model, and cosine similarity was used to identify the three most similar patients to each query patient from a database of embeddings derived from 2 years of cardiology admissions. A total of 25 query patients and 75 retrieved patients were evaluated. The list of similar patients and their hospital trajectories was retrieved and presented for each submitted query case. The model results were presented on the day of admission of the query patients. The clinical utility rankings for the model outcomes were also reassessed 2 weeks post-admission. The objective of this 2-week post-admission evaluation was to determine whether the clinician’s perceived usefulness of the retrieved patient similarities had changed over time as their knowledge of the query patients evolved. Our clinical validation approaches align with published frameworks on trustworthy AI deployment, ensuring transparency, reliability, and real-world applicability, as outlined in the FUTURE-AI framework, an international consensus guideline^67^.

### Analysis platforms

All data analyses, along with modelling, were performed using the KubeFlow^68^ and Aridhia Data Research Environment^69^ platforms. The libraries used included Python v3.9^70^, Pandas v2.1.1^71^, Numpy v1.24.4^72^, Scikit-Learn v1.6.1^73^, SciPy v1.11.3^74^, PyTorch v2.6.0+cu124^75^, Pingouin v0.5.5^76^, and the Hugging Face Transformers library v4.49.0^77^.

## Supplementary information

Supplementary Information

Transparent Peer Review file

## Source data

Source data

## Data Availability

The data supporting the findings from this study are available within the manuscript and its supplementary information. Source data are provided with this paper. The GOSH clinical dataset generated in this study are not deposited in a public database due to patient privacy and governance restrictions. The code used for modelling GOSH data has been developed on MIMIC-IV for reproducibility, and can be accessed via the link provided in the code availability statement. The raw GOSH data are protected and are not publicly available due to data privacy laws. Access to anonymised data can be requested through Great Ormond Street Hospital (GOSH) Data Research, Innovation and Virtual Environments (DRE), subject to appropriate research approvals (more information available at: https://www.gosh.nhs.uk/our-research/drive-unit-for-digital-innovation/a-secure-environment-fpr-digital-research/. The request form to register interest in accessing the data is available at https://forms.monday.com/forms/2d6d654cd1af45526a14a758d3f3c7f3?r=euc1. The MIMIC-IV dataset used in this study is publicly available in the PhysioNet database under open access (https://physionet.org/content/mimiciv/3.1/)^50^. Access requires completion of the CITI ‘Data or Specimens Only Research’ training, agreement to the PhysioNet Data Use Agreement, and registration for a PhysioNet account. After completing these steps, users are typically granted access within 1–2 business days. The dataset may be used for research purposes only, and redistribution or commercial use is prohibited under the terms of the agreement. Source data are provided with this paper.

## Code availability

The code for the MIMIC models can be found at https://github.com/tmi-lab/GOSH-EHR-cardiology.
