# Supplementary material for: Clinically-applicable prediction of hospital stay and patient similarity retrieval in paediatric cardiology using machine learning
Source: Nat Commun. 2026 May 13;17:6370. doi: 10.1038/s41467-026-73021-3 (PMC13376627; doi:10.1038/s41467-026-73021-3)
Supplement: Supplementary file 1 — Supplementary Information [file 41467_2026_73021_MOESM1_ESM.pdf]

Supplementary Information

Supplementary Table 1: Performance metrics for models trained and evaluated only on the MIMIC-IV dataset. Accuracy represents the overall accuracy across both short and long stay.

LoS	**Short Stay**				**Long Stay**				
	Sensitivity	Specificity	Precision	F1 Score	Sensitivity	Specificity	Precision	F1 Score	Accuracy
BioClinical BERT (FT-MIMIC)	0.52 ± 0.01	0.87 ± 0.01	0.85 ± 0.01	0.64 ± 0.01	0.87 ± 0.01	0.52 ± 0.01	0.55 ± 0.01	0.68 ± 0.01	0.66 ± 0.01
ROBERTA (FT-MIMIC)	0.50 ± 0.01	0.86 ± 0.01	0.84 ± 0.00	0.62 ± 0.01	0.86 ± 0.01	0.50 ± 0.01	0.54 ± 0.01	0.67 ± 0.01	0.65 ± 0.00
LSTM (FT-MIMIC)	0.15 ± 0.00	0.89 ± 0.00	0.67 ± 0.01	0.24 ± 0.01	0.89 ± 0.00	0.15 ± 0.00	0.42 ± 0.00	0.57 ± 0.00	0.45 ± 0.00

FT-{x}; Fine-tuned with dataset from x, LoS; Length of stay.

Supplementary Table 2: Length of Stay Performance Metrics for Sex across Folds (Random Forest).

Sex	LoS (n)	Precision	Recall	F1	Specificity	Accuracy
Female	Short Stay (33)	0.672 ± 0.072	0.825 ± 0.066	0.738 ± 0.052	0.866 ± 0.036	0.855 ± 0.031
	Long Stay (97)	0.936 ± 0.028	0.866 ± 0.036	0.899 ± 0.025	0.825 ± 0.066	0.855 ± 0.031
Male	Short Stay (49)	0.781 ± 0.059	0.769 ± 0.056	0.773 ± 0.045	0.915 ± 0.025	0.873 ± 0.024
	Long Stay (123)	0.909 ± 0.024	0.915 ± 0.025	0.911 ± 0.018	0.769 ± 0.056	0.873 ± 0.024

LoS; Length of Stay.

Supplementary Table 3: Length of Stay Performance Metrics for Different Ethnicities Across Folds (Random Forest).

Ethnicity	LoS (n)	Precision	Recall	F1	Specificity	Accuracy
Asian	Short Stay (13)	1.000 ± 0.000	0.621 ± 0.133	0.757 ± 0.110	1.000 ± 0.000	0.907 ± 0.043
	Long Stay (40)	0.890 ± 0.050	1.000 ± 0.000	0.941 ± 0.029	0.621 ± 0.133	0.907 ± 0.043
Black	Short Stay (13)	0.865 ± 0.102	0.869 ± 0.087	0.862 ± 0.071	0.912 ± 0.068	0.894 ± 0.050
	Long Stay (20)	0.913 ± 0.056	0.912 ± 0.068	0.910 ± 0.046	0.869 ± 0.087	0.894 ± 0.050
Mixed	Short Stay (4)	0.822 ± 0.183	1.000 ± 0.000	0.890 ± 0.125	0.944 ± 0.061	0.956 ± 0.046
	Long Stay (17)	1.000 ± 0.000	0.944 ± 0.061	0.970 ± 0.034	1.000 ± 0.000	0.956 ± 0.046
Other	Short Stay (2)	0.412 ± 0.379	0.465 ± 0.415	0.411 ± 0.359	0.945 ± 0.052	0.905 ± 0.070
	Long Stay (19)	0.949 ± 0.057	0.945 ± 0.052	0.946 ± 0.043	0.465 ± 0.415	0.905 ± 0.070
White	Short Stay (34)	0.636 ± 0.067	0.894 ± 0.047	0.741 ± 0.050	0.806 ± 0.042	0.830 ± 0.031
	Long Stay (88)	0.952 ± 0.022	0.806 ± 0.042	0.873 ± 0.026	0.894 ± 0.047	0.830 ± 0.031

LoS; Length of Stay.

Supplementary Table 4: Length of Stay Performance Metrics for Sex across Folds (BioClinical-BERT).

Sex	LoS (n)	Precision	Recall	F1	Specificity	Accuracy
Female	Short Stay (38)	0.787 ± 0.168	0.789 ± 0.191	0.777 ± 0.145	0.948 ± 0.034	0.789 ± 0.191
	Long Stay (97)	0.929 ± 0.046	0.948 ± 0.034	0.938 ± 0.031	0.789 ± 0.191	0.948 ± 0.034
Male	Short Stay (49)	0.830 ± 0.122	0.795 ± 0.157	0.797 ± 0.076	0.928 ± 0.062	0.795 ± 0.157
	Long Stay (123)	0.913 ± 0.064	0.928 ± 0.062	0.918 ± 0.037	0.795 ± 0.157	0.928 ± 0.062

LoS; Length of Stay.

Supplementary Table 5: Length of Stay Performance Metrics for Different Ethnicities Across Folds (BioClinical-BERT).

Ethnicity	LoS (n)	Precision	Recall	F1	Specificity	Accuracy
Asian	Short Stay (13)	0.800 ± 0.447	0.620 ± 0.415	0.683 ± 0.410	1.000 ± 0.000	0.620 ± 0.415
	Long Stay (40)	0.887 ± 0.118	1.000 ± 0.000	0.937 ± 0.068	0.620 ± 0.415	1.000 ± 0.000
Black	Short Stay (13)	0.933 ± 0.149	0.833 ± 0.236	0.867 ± 0.183	0.960 ± 0.089	0.833 ± 0.236
	Long Stay (20)	0.920 ± 0.110	0.960 ± 0.089	0.938 ± 0.091	0.833 ± 0.236	0.960 ± 0.089
Mixed	Short Stay (4)	0.800 ± 0.447	0.800 ± 0.447	0.800 ± 0.447	1.000 ± 0.000	1.000 ± 0.000
	Long Stay (17)	1.000 ± 0.000	1.000 ± 0.000	1.000 ± 0.000	1.000 ± 0.000	1.000 ± 0.000
Other	Short Stay (2)	0.200 ± 0.447	0.200 ± 0.447	0.200 ± 0.447	0.933 ± 0.149	0.500 ± 0.707
	Long Stay (19)	0.950 ± 0.112	0.933 ± 0.149	0.931 ± 0.096	0.200 ± 0.447	0.933 ± 0.149
White	Short Stay (34)	0.678 ± 0.216	0.842 ± 0.289	0.745 ± 0.236	0.878 ± 0.052	0.842 ± 0.289
	Long Stay (88)	0.966 ± 0.048	0.878 ± 0.052	0.918 ± 0.026	0.842 ± 0.289	0.878 ± 0.052

LoS; Length of Stay.

Supplementary Table 6: Confusion matrices for (a) BioClinical BERT MIMIC-GOSH and (b) Random Forest for the GOSH LoS classification task.

	Pred Short Stay	Pred Long Stay
True Short Stay	65	17
True Long Stay	18	202

(a) BioClinical BERT MIMIC-GOSH

	Pred Short Stay	Pred Long Stay
True Short Stay	64	18
True Long Stay	14	206

(b) Random Forest

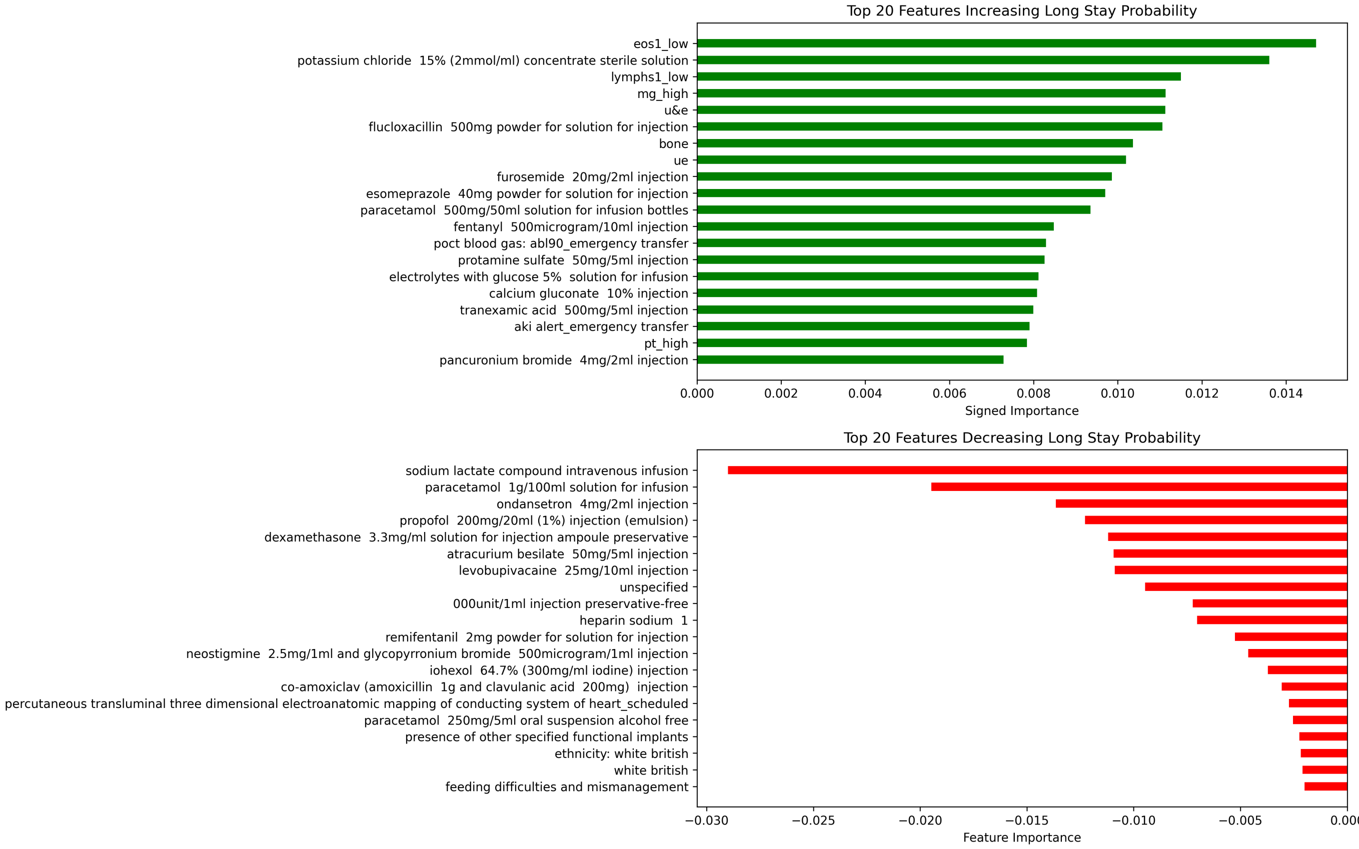

Supplementary Figure 1: Top Text Features Influencing Predicted Length of Stay. Top 20 text-derived features from the random-forest model that (left) increase and (right) decrease the predicted probability of a prolonged hospital stay. Bars show signed importances; positive values (green) increase the probability of long-stay, while negative values (red) lower it. Source data are provided as a Source Data file.

Supplementary Table 7: Performance metrics (precision, recall, F1, specificity, accuracy) for the threshold-optimised traffic-light system and comparison models.

Model	Precision	Recall	F1	Specificity	Accuracy
BioClinical BERT MIMIC-GOSH	0.909	0.952	0.930	0.778	0.900
Random Forest	0.955	0.859	0.905	0.890	0.868

Terms such as “U&E,” “bone,” “lymphs low,” “magnesium high,” “eosinophils low,” “platelets high,” and medications including “tranexamic acid,” “flucloxacillin,” “fentanyl,” “potassium chloride,” “furosemide,” “esomeprazole,” and “calcium gluconate” may signal electrolyte imbalance, infection, inflammation, or acute intervention. These may be markers of higher acuity that often are associated with prolonged hospital admissions. In contrast, terms such as “sodium lactate infusion,” “levobupivacaine,” “ondansetron,” “paracetamol,” “atracurium,” and “propofol,” as well as generic descriptors like “unspecified,” “white British,” and “feeding difficulties and mismanagement” tend to appear in perioperative care, routine symptom management, or standardised treatment protocols, which are contexts typical of lower-severity or more controlled clinical pathways that may result in quicker discharge, and shorter overall hospital stays. These associations are correlational, not causal.

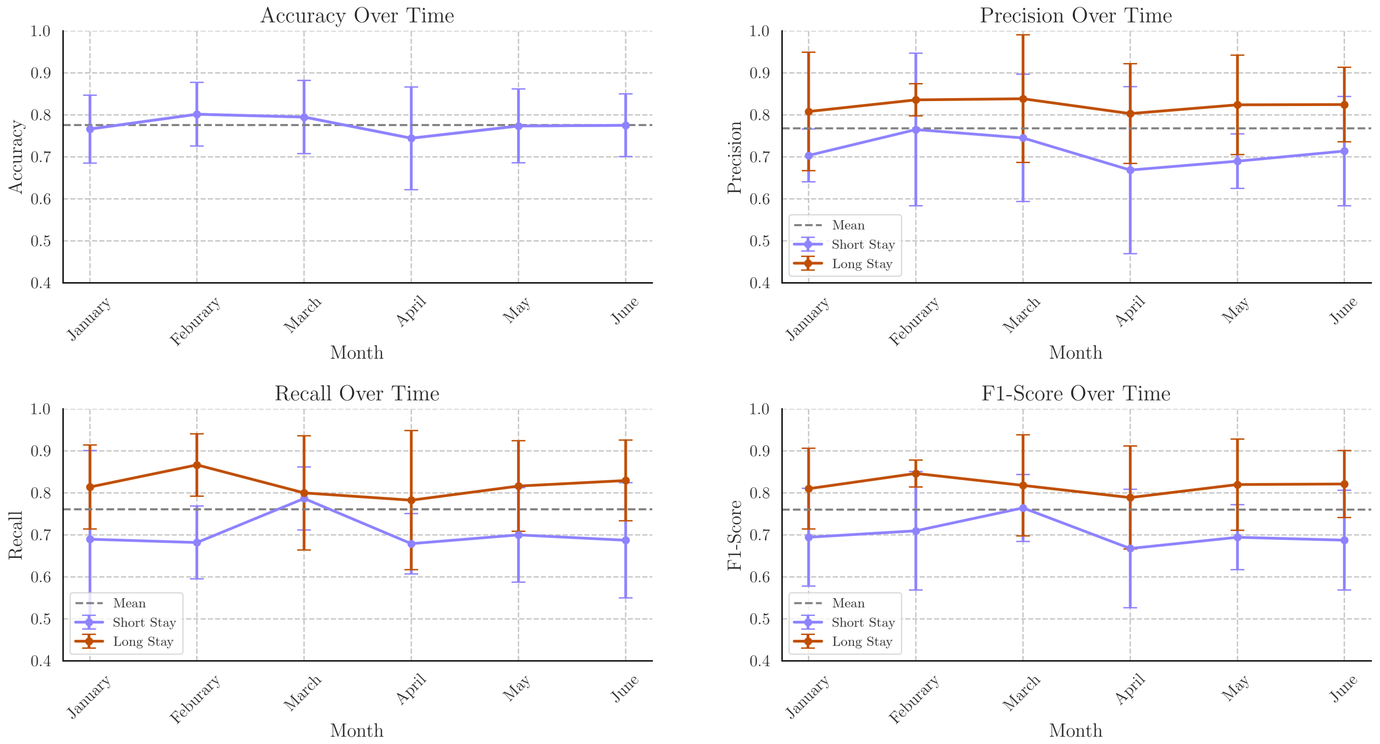

Supplementary Figure 2: Silent deployment performance (BioClinical-BERT).
Panels show performance metrics (a) accuracy, b) precision, c) recall, and d) F1-score) aggregated by one-month intervals across 01/01/24 - 31/06/24. Monthly sample sizes were: January (n=218), February (n=161), March (n=166), April (n=184), May (n=181), and June (n=142). Error bars represent the distribution of recall values across patients within each month. The grey dotted line represents the mean value across the six-month period. Source data are provided as a Source Data file.

The MIMIC-trained, GOSH fine-tuned binary classification BERT model for predicting patient Length of Stay (LOS) (short stay, long stay) was deployed within the Digital Research Environment (DRE) unit at GOSH, processing real-time data from admissions over a six-month period. The results indicate an overall accuracy of 0.76 (± 0.02). For Short Stay metrics, mean precision was 0.71 (± 0.04), recall was 0.70 (± 0.04), and F1-score was 0.70 (± 0.03). In contrast, Long Stay metrics showed mean precision of 0.82 (± 0.01), recall of 0.82 (± 0.03), and F1-score of 0.82 (± 0.02). The average revealed a precision of 0.77 (± 0.03), recall of 0.61 (± 0.04), and F1-score of 0.76 (± 0.03). Supplementary Table 10 reports the

selected demographic and statistical information for pre-processed Silent Deployment GOSH monthly data. Supplementary Figure 2 illustrates the BERT model's performance between 01/01/24 - 31/06/24.

Query patient 1: Male, Age: 7 days, coarctation aorta, coractation repair – emergency, fbc; normal. u+e; high bilirubin, dinoprostone 5ng/k/min IV infusion, coamoxiclav 96mg IV, clonidine 1mcg/k/min IV infusion, morphine 18mcg/k/h IV infusion. adrenaline 0.03 mcg/k/min IV infusion, milrinone 0.2mcg/k/h IV infusion.

(a) Query patient submission example

Similar Patient 1 – High Similarity (score: 0.86)

The patient, Female, Age: 16 months, was admitted as an inpatient under cardiology via emergency transfer, and discharged on clinical advice/consent, after a 48.17-hour stay (2 days), with an unspecified principal problem. The admission was associated with NHS - Ward for General Patients, Younger Physically Disabled.

Day	Medical Events	Summary
1	dinoprostone continuous infusion 50mcg/50ml pre-filled syringe, potassium chloride 0.15% (10mmol potassium) in glucose 10% and sodium chloride 0.45% 500ml infusion, po5_high, urea - high, create - low, eos1_high, lymphs1 - low, mchc1 - high, wbc1 - low, tt - high, other specified: plastic repair of aorta scheduled, Ventricular septal defect	Medication administered: elevated phosphate and urea levels; low creatinine; abnormalities in blood counts and MCHC; scheduled plastic repair of aorta; diagnosis of ventricular septal defect.
2	transthoracic echocardiogram, u+e, bone, lft, aki alert, poct blood gas: abl90, bilirubin (split), transthoracic echocardiography, radiology of one body area (or < 20 minutes), heparin cvl flush 1 unit/ml (50 units/50ml), create -low, Ventricular septal defect, Observation for other suspected cardiovascular diseases, Coarctation of aorta, Other congenital malformations of aorta	Emergency procedures: Echocardiogram and radiology; tests for kidney function, bilirubin levels, and blood gases; heparin flush; monitoring and observation for cardiovascular conditions, including aortic malformations and coarctation.

(b) Retrieved similar patient example

Supplementary Figure 3: Example of Patient Case Retrieval. a) A sample of a query patient along with (b) a corresponding prediction for a similar patient and their hospital trajectory based on the highest cosine similarity between the query patients vector and a database of embeddings from prior patients. Demographic information, primary diagnoses and dates have been removed from these examples to protect data privacy.

A regression-based BioClinical-BERT model was developed to predict length of stay (LOS) as a single numeric value by modifying the original BioClinical-BERT architecture to include a regression head. The model's performance was evaluated using Mean Absolute Error (MAE) across 5-fold cross-validation for different day intervals. The regression based model demonstrated a MAE of 3.73 ($\pm$ 1.0801) for the 1-4 day range, 6.42 ($\pm$ 1.6801) for the 5-8 days, 7.27 ($\pm$ 0.7254) for 9-12 days and 7.01 ($\pm$ 0.1203) for the 13-17 days. The distribution of actual vs. predicted values (Supplementary Figure 2) shows an overestimation of predicted values in higher ranges compared to actual values, particularly beyond 6 days. This suggests the regression-based model performs well for short-term predictions but struggles with longer-term forecasts.

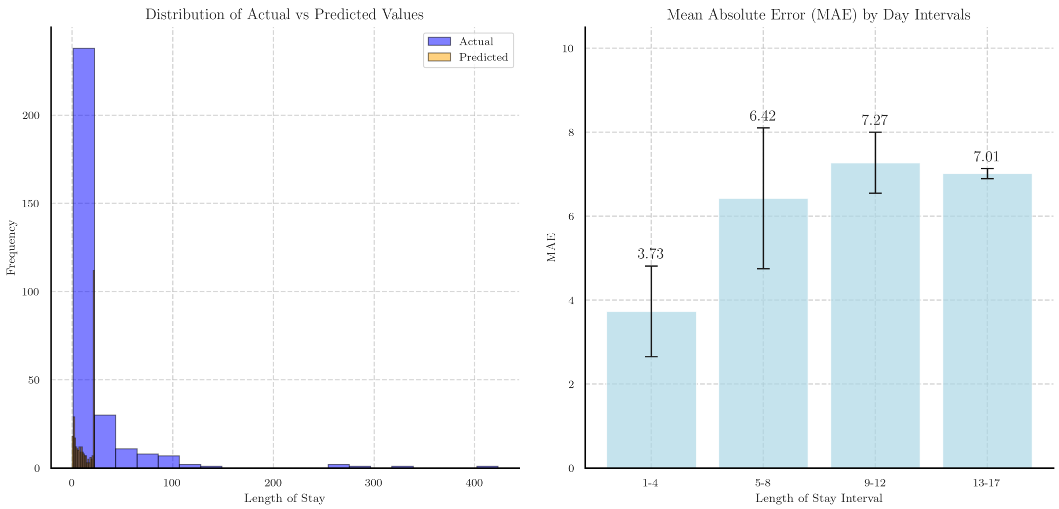

Supplementary Figure 4: Performance and Error of BioClinical-BERT Length-of-Stay Predictions. Distribution and performance metrics (MAE) of the regression BioClinical-BERT model, fine-tuned on the MIMIC dataset and subsequently on the GOSH dataset. Panel a) depicts the distribution of true labels against the distribution of model-predicted values, while panel b) presents test-set MAE across different lengths of stay interval predictions (1-4, 5-8, 9-12, 13-17). Error bars represent standard deviation across 5 k-validation folds. MAE; Mean absolute error. Source data are provided as a Source Data file.

Supplementary Table 8: Demographic Categories for GOSH and MIMIC-IV Datasets.

Category	GOSH	MIMIC-IV
White	White British, Other White Background, White Irish	White-E. European, White-Russian, White-Brazilian, White-Portuguese
Black	Black African, Black Caribbean, Other Black Background	Black-African American, Black-Caribbean, Black-Cape Verdean
Asian	Asian Pakistani, Asian Indian, Other Asian Background, Asian Bangladeshi, Other Chinese	Asian, Asian-Chinese, Asian-South East Asian, Asian-Indian, Asian-Korean
Mixed	Mixed White and Asian, Mixed White and Black Caribbean, Mixed White and Black African, Other Mixed Background	Multiple Race/Ethnicity
Other	Other Ethnic Backgrounds, Prefer Not to Say, N/A	Alaska Native, Native Hawaiian or Pacific Islander, Other, Hispanic/Latino
Not Available	N/A	Unknown, Unable to Obtain, Patient Declined

Supplementary Table 9: Model parameters and key specifications for BioClinicalBERT.

Parameter	Value
Model Architecture	Bidirectional Encoder Representations from Transformers (BERT)
Model Variant	BioClinicalBERT
Pre-training Corpus	MIMIC-III, MIMIC-IV
Vocabulary Size	30,522 tokens
Number of Layers	12
Hidden Size	768
Number of Attention Heads	12
Intermediate Size	3,072
Activation Function	GELU
Dropout Rate	0.1
Number of Parameters	$\sim$110M
Sequence Length	512 tokens (max)
Optimizer	AdamW
Learning Rate	2×10^{-5} (fine-tuning)
Batch Size	16-32 (fine-tuning)
Pre-training Objective	Masked Language Modelling (MLM) & Next Sentence Prediction (NSP)

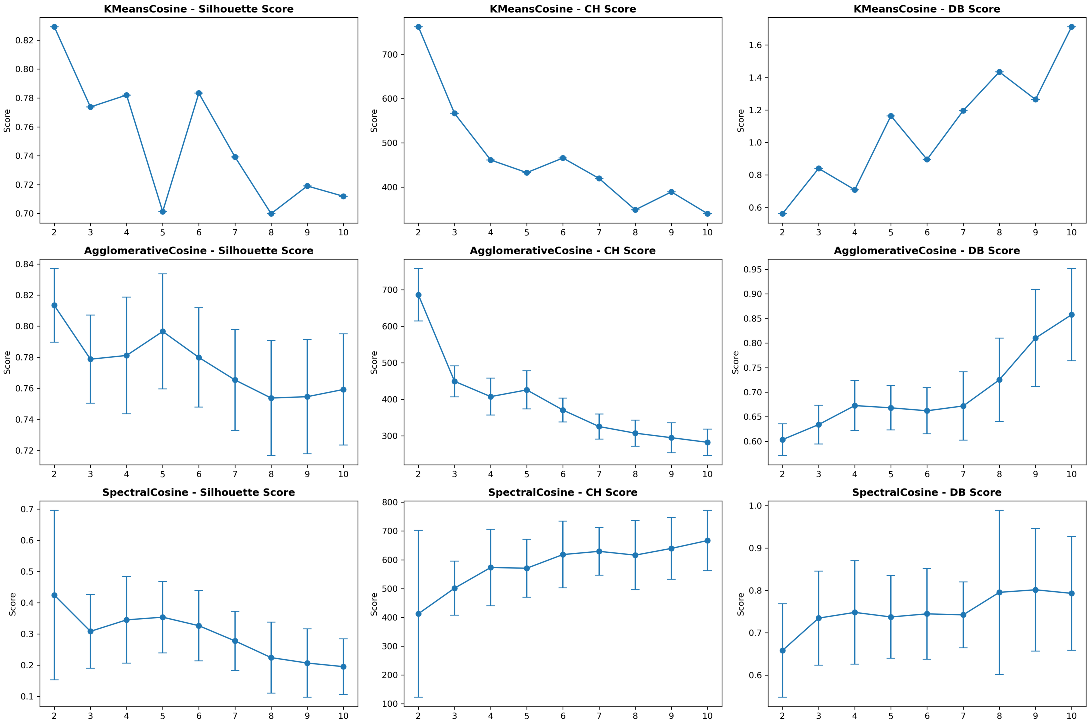

Supplementary Figure 5: Optimal Cluster Selection Across Clustering Algorithms. Optimal cluster determination using Silhouette score, Davies-Bouldin, and Calinski-Harabasz Indices for K-Means, Agglomerative and Spectral clustering algorithms, across 10 iterations. Source data are provided as a Source Data file.

CH; Calinski-Harabasz, DB; Davies Bouldin, Dist; distance

Supplementary Table 10: Performance metrics for evaluation of K-Means, Agglomerative and Spectral clustering with cosine distance across 10 runs. Higher SC and CH scores indicate better clustering performance, whilst a lower DB score indicates better performance.

Model	Clusters (n)	Silhouette score	CH Index	DB Index
KMeansCosine	2	0.829330 ± 0.00	762.707956 ± 0.00	$0.561873 \pm 2.22 \times 10^{-16}$
	3	$0.773754 \pm 5.96 \times 10^{-8}$	566.689824 ± 0.00	$0.840253 \pm 2.22 \times 10^{-16}$
	4	$0.782031 \pm 1.79 \times 10^{-7}$	$461.211728 \pm 5.68 \times 10^{-14}$	0.708666 ± 0.00
	5	$0.701354 \pm 1.19 \times 10^{-7}$	$432.670837 \pm 5.68 \times 10^{-14}$	1.163979 ± 0.00
	6	$0.783318 \pm 5.96 \times 10^{-8}$	$465.744777 \pm 2.84 \times 10^{-13}$	$0.895393 \pm 3.33 \times 10^{-16}$
	7	$0.739186 \pm 1.19 \times 10^{-7}$	$419.444932 \pm 5.68 \times 10^{-14}$	$1.195922 \pm 4.44 \times 10^{-16}$
	8	$0.699887 \pm 2.38 \times 10^{-7}$	$348.399267 \pm 5.68 \times 10^{-14}$	$1.434333 \pm 4.44 \times 10^{-16}$
	9	$0.719051 \pm 1.19 \times 10^{-7}$	$389.098301 \pm 1.14 \times 10^{-13}$	1.264723 ± 0.00
	10	$0.711920 \pm 5.96 \times 10^{-8}$	339.805721 ± 0.00	$1.712584 \pm 8.88 \times 10^{-16}$
AgglomerativeCosine	2	$0.813392 \pm 2.37 \times 10^{-2}$	$686.185318 \pm 7.19 \times 10^{1}$	$0.603462 \pm 3.21 \times 10^{-2}$
	3	$0.778768 \pm 2.83 \times 10^{-2}$	$449.310846 \pm 4.24 \times 10^{1}$	$0.634079 \pm 3.95 \times 10^{-2}$
	4	$0.781149 \pm 3.75 \times 10^{-2}$	$407.425557 \pm 5.06 \times 10^{1}$	$0.672714 \pm 5.09 \times 10^{-2}$
	5	$0.796607 \pm 3.70 \times 10^{-2}$	$425.827329 \pm 5.22 \times 10^{1}$	$0.668151 \pm 4.50 \times 10^{-2}$
	6	$0.779895 \pm 3.19 \times 10^{-2}$	$370.617423 \pm 3.26 \times 10^{1}$	$0.662171 \pm 4.69 \times 10^{-2}$
	7	$0.765400 \pm 3.24 \times 10^{-2}$	$325.508057 \pm 3.42 \times 10^{1}$	$0.671916 \pm 6.95 \times 10^{-2}$
	8	$0.753771 \pm 3.69 \times 10^{-2}$	$307.455069 \pm 3.59 \times 10^{1}$	$0.725102 \pm 8.47 \times 10^{-2}$
	9	$0.754638 \pm 3.68 \times 10^{-2}$	$295.029875 \pm 4.10 \times 10^{1}$	$0.810039 \pm 9.90 \times 10^{-2}$
	10	$0.759290 \pm 3.57 \times 10^{-2}$	$282.406702 \pm 3.59 \times 10^{1}$	$0.857665 \pm 9.39 \times 10^{-2}$
SpectralCosine	2	$0.424386 \pm 2.72 \times 10^{-1}$	$412.649280 \pm 2.90 \times 10^{2}$	$0.658371 \pm 1.10 \times 10^{-1}$
	3	$0.308277 \pm 1.18 \times 10^{-1}$	$501.396053 \pm 9.39 \times 10^{1}$	$0.734666 \pm 1.11 \times 10^{-1}$
	4	$0.345083 \pm 1.39 \times 10^{-1}$	$573.246182 \pm 1.33 \times 10^{2}$	$0.748185 \pm 1.22 \times 10^{-1}$
	5	$0.353673 \pm 1.14 \times 10^{-1}$	$570.747699 \pm 1.01 \times 10^{2}$	$0.737389 \pm 9.76 \times 10^{-2}$
	6	$0.326384 \pm 1.13 \times 10^{-1}$	$618.276960 \pm 1.16 \times 10^{2}$	$0.744746 \pm 1.07 \times 10^{-1}$
	7	$0.277614 \pm 9.47 \times 10^{-2}$	$629.455685 \pm 8.26 \times 10^{1}$	$0.742355 \pm 7.79 \times 10^{-2}$
	8	$0.224165 \pm 1.14 \times 10^{-1}$	$616.270469 \pm 1.20 \times 10^{2}$	$0.795493 \pm 1.94 \times 10^{-1}$
	9	$0.206833 \pm 1.09 \times 10^{-1}$	$639.557864 \pm 1.07 \times 10^{2}$	$0.801493 \pm 1.45 \times 10^{-1}$
	10	$0.195262 \pm 8.89 \times 10^{-2}$	$666.949084 \pm 1.05 \times 10^{2}$	$0.793100 \pm 1.34 \times 10^{-1}$

CH; Calinski-Harabasz, DB; Davies Bouldin

The optimal number of clusters for the GOSH BioClinical-BERT trained embeddings was determined by first scaling the input data and reducing its dimensionality to three dimensions using t-SNE. K-Means, Agglomerative and spectral clustering algorithms were fitted using cosine distance across a range of cluster numbers $k \in [2, 10]$. KMeansCosine was selected at k = 3 based on clustering quality and stability in 10 iterations with 100 bootstrap samples, as well as clinical interpretability. At k=3, the Silhouette score (0.77) and CH score were high, indicating well-separated and compact clusters, while the DB score remained low, suggesting good cluster compactness. Although k=2 achieved the highest metric values, k=3 was selected as it provides greater granularity and captures additional clinically relevant substructure that may be obscured when the data are partitioned into only two groups. Additionally, the relatively small error bars across all metrics demonstrate the stability of cluster assignments, supporting k=3 as the optimal choice for the GOSH-trained embeddings.

Supplementary Table 11: Patient Profiling of Clusters.

Cluster	Sex (% of cluster)	Age (95% CI)	Mean LoS (95% CI)	Complication rate (%) (95% CI)	Emergency rate (%) (95% CI)	Mortality rate (%) (95% CI)	Ethnicity (%)
1	M: 46.43%<br>F: 53.57%	8.08<br>(7.02, 9.33)	24.47<br>(16.0, 34.58)	15.84%<br>(10.89, 20.79)	22.73%<br>(13.64, 33.33)	1.39%<br>(0.35, 2.79)	White: 38.09%<br>Asian: 23.80%<br>Black: 6.79%<br>Mixed/Other: 31.32%
2	M: 57.55%<br>F: 42.45%	10.31<br>(9.47, 11.26)	13.76<br>(7.45, 22.01)	17.82%<br>(12.87, 23.27)	21.21%<br>(12.12, 31.82)	1.05%<br>(0.00, 2.44)	White: 46.76%<br>Asian: 9.36%<br>Black: 9.63%<br>Mixed/Other: 34.25%
3	M: 68.92%<br>F: 31.08%	6.24<br>(5.17, 7.30)	30.09<br>(22.51, 40.30)	13.37%<br>(8.91, 18.32)	36.36%<br>(25.76, 48.48)	1.05%<br>(0.00, 2.44)	White: 33.78%<br>Asian: 17.57%<br>Black: 8.11%<br>Mixed/Other: 40.54%

CI; Confidence interval, F; Female, LoS; Length of stay, M; Male.

Supplementary Table 12: Pairwise comparisons of mean LoS between clusters using a two-sided Tukey HSD test with family-wise error rate correction (FWER = 0.05) following one-way ANOVA.

group1	group2	meandiff	p-adj	lower	upper	reject
1	2	-10.71	0.199	-25.36	3.94	False
1	3	5.63	0.71	-11.28	22.53	False
2	3	16.33	0.03	1.08	31.59	True

Supplementary Table 13: Cluster-level counts and proportions of emergency events. Pairwise comparisons between clusters were performed using two-sided Fisher's exact tests; reported p-values are unadjusted for multiple comparisons.

Cluster	Count	Proportion Percentage	Fisher's Test p-value
1	15	28.30	C1-C2: 0.3817
2	14	26.52	C1-C3: 0.6284
3	24	45.28	C2-C3: 0.8235

Ci–Cj: significance between cluster i and j.

Supplementary Table 14: Cluster-level counts and proportions of complications. Pairwise comparisons between clusters were performed using two-sided Fisher's exact tests; reported p-values are unadjusted for multiple comparisons.

Cluster	Count	Proportion Percentage	Fisher's Test p-value
1	32	33.68	C1-C2: 0.1169
2	36	37.89	C1-C3: 0.0714
3	27	28.42	C2-C3: 0.8702

Ci–Cj: significance between cluster i and j.

Supplementary Table 15: Cluster-level counts and proportions of deaths. Pairwise comparisons between clusters were performed using two-sided Fisher's exact tests; reported p-values are unadjusted for multiple comparisons.

Cluster	Count	Proportion Percentage	Fisher's Test p-value
1	4	40.0	C1-C2: 0.4208
2	3	30.0	C1-C3: 0.4299
3	3	30.0	C2-C3: 1.0

Ci–Cj: significance between cluster i and j.

Supplementary Table 16: Pairwise comparisons of mean age between clusters using a two-sided Tukey HSD test with family-wise error rate correction (FWER = 0.05) following one-way ANOVA.

Group 1	Group 2	Mean Diff	p-adj	Lower	Upper	Reject
1	2	2.2214	0.01	0.44	4.00	True
1	3	-1.88	0.08	-3.9388	0.1726	False
2	3	-4.1045	0.0	-5.9600	-2.2490	True

Supplementary Table 17: Selected demographic and statistical information for pre-processed Silent Deployment GOSH monthly data.

	January	February	March	April	May	June
Patients	218	161	166	184	181	142
Male	122 (55.9%)	89 (55.2%)	99 (59.6%)	103 (55.9%)	93 (51.3%)	77 (54.2%)
Female	96 (44.1%)	72 (44.8%)	67 (41.6%)	81 (44.1%)	90 (49.7%)	67 (46.8%)
Age						
Min	0.70	0.60	0.50	0.40	0.30	0.30
Max	19.00	18.50	18.80	18.30	18.10	18.40
Mean ± SD	7.10 ± 5.53	6.39 ± 5.46	6.94 ± 5.90	7.27 ± 5.63	6.56 ± 5.54	6.49 ± 5.62
White	95 (43.5%)	70 (43.4%)	90 (54.2%)	81 (44.0%)	85 (43.4%)	63 (44.3%)
Black	25 (11.4%)	8 (4.9%)	12 (7.2%)	12 (6.5%)	9 (4.9%)	8 (5.6%)
Asian	32 (14.6%)	26 (16.1%)	16 (9.6%)	22 (11.9%)	33 (18.2%)	23 (16.1%)
Mixed	7 (3.2%)	6 (3.7%)	6 (3.6%)	17 (9.2%)	9 (4.9%)	5 (3.5%)
Other	54 (24.7%)	51 (26.7%)	42 (25.3%)	43 (23.3%)	44 (24.3%)	45 (31.6%)
Short stay	84 (38.5%)	56 (37.8%)	71 (42.8 %)	69 (37.5 %)	67 (37.0 %)	54 (38.0 %)
Long stay	134 (61.5%)	105 (65.2%)	95 (57.2%)	115 (62.5%)	114 (63.0%)	88 (62.0%)

N/A; Not Available, SD; standard deviation.
