# Supplementary material for: Clinically-applicable prediction of hospital stay and patient similarity retrieval in paediatric cardiology using machine learning
Source: Nat Commun. 2026 May 13;17:6370. doi: 10.1038/s41467-026-73021-3 (PMC13376627; doi:10.1038/s41467-026-73021-3)
Supplement: Supplementary file 2 — Transparent Peer Review file [file 41467_2026_73021_MOESM2_ESM.pdf]

nature portfolio

Peer Review File

Clinically-applicable prediction of hospital stay and patient similarity retrieval in paediatric cardiology using machine learning

Corresponding Author: Professor Payam Barnaghi

This file contains all reviewer reports in order by version, followed by all author rebuttals in order by version.

Version 0:

Reviewer comments:

Reviewer #5

(Remarks to the Author)
Thank you for the opportunity to review this manuscript. The authors present a timely and technically ambitious study leveraging machine learning (ML) methods for predicting length of hospital stay (LoS) and retrieving similar patient cases in paediatric cardiology. This area of research is particularly valuable given the complexity and heterogeneity of congenital heart disease and other pediatric cardiac conditions. The manuscript demonstrates solid innovation and implementation rigor, though there are areas that require further clarification, refinement, and discussion before publication.
Narrative Structure, Grammar, and Flow
The manuscript is generally well-written and adheres to standard academic structure. The flow of ideas is logical, with a smooth transition between sections. However, some sentences in the Abstract and Discussion sections are densely packed with multiple technical elements and could benefit from clearer segmentation for reader comprehension. Additionally, a few instances of passive voice and overly complex phrasing may hinder clarity for multidisciplinary audiences.
1. Introduction
The introduction clearly outlines the motivation for applying ML in paediatric cardiology, where clinical complexity, data sparsity, and unpredictability in disease trajectories pose significant challenges. However, the current version would benefit from:
• A deeper review of previous work on ML for LoS prediction and patient similarity in paediatric or adult populations, including any limitations.
• An explicit gap statement explaining what this study does that prior studies did not—especially in terms of real-world clinical deployment.
2. Rationale and Novelty
The integration of LoS prediction with case similarity retrieval, and benchmarking against clinician performance, reflects commendable novelty. Silent deployment within the NHS and pilot feedback from clinicians further enhance translational relevance. This dual utility (predictive + retrieval) framework is innovative, though the underlying conceptual link between these two tasks could be made more explicit in the Methods and Discussion.
3. Methodology
The methodological pipeline is broadly appropriate and adheres to contemporary standards in clinical ML studies. Strengths include:
• Use of BioClinical-BERT for semantic embedding extraction.
• External comparison with clinicians as a reference standard.
• Real-world deployment in a large NHS system.
However, several methodological concerns need clarification:
• Data Quality & Missingness: There is no detailed account of missing data handling, imputation strategies, or data completeness across EHR variables. Were time-series features complete for all 1,002 patients?
• Feature Engineering: It is unclear which structured or unstructured features were used, how they were processed (e.g., one-hot encoding, normalization), and whether any selection techniques were applied.
• Timeline Alignment: The process of aligning patient records to a 24-hour timeline is mentioned briefly. This step warrants a fuller description—what is the unit of aggregation? How was temporal sparsity handled?
• Model Interpretability: While RF models are generally interpretable, there is no report of feature importance, SHAP values, or clinical reasoning correlates—key for clinical trust.

• Similarity Retrieval Evaluation: How was the retrieval performance quantified beyond anecdotal clinician feedback? Was there any ranking metric like MRR, NDCG, or qualitative rating scale applied to top-k matches?
• Data Partitioning: The strategy for train/test split, cross-validation, and time-based validation (if any) is unclear.
Figures and Tables
• Ensure clear and labeled confusion matrices or ROC curves for LoS prediction.
• Include representative retrieval results or embedding cluster visualizations (e.g., t-SNE/UMAP).
• Consider adding clinician-level performance variability and model confidence stratification.
• Tables should compare model metrics vs. clinician estimates over the same cohort.
4. Results and Observations
The results are compelling, particularly the LoS prediction performance (accuracy 0.91 ± 0.01) and the reported superiority over clinicians ($p < 0.001$). Additional strengths include:
• High precision and sensitivity metrics across six months of silent deployment.
• Identification of three clinically distinct subgroups through unsupervised clustering.
• Inclusion of subjective clinician feedback in real clinical settings.
However, results could be strengthened by:
• Disaggregating model performance by key patient subgroups (age, primary diagnosis, comorbidities).
• Reporting misclassification rates and common sources of error.
• Providing comparative performance of all tested models—not just RF.
5. Discussion
The Discussion appropriately highlights the clinical relevance and future potential of ML-based prediction and retrieval systems. Nonetheless, a few enhancements are needed:
• Limitations: The authors do not fully engage with limitations such as generalizability beyond GOSH, algorithmic bias in small datasets, or the risks of overfitting in niche populations.
• Ethical Considerations: There is no mention of ethical oversight, potential for automation bias, or plans for integrating human–AI collaboration in decision-making.
• Scalability: How transferable is the model to other NHS settings or international hospitals with different EHR systems?
6. Other Minor Comments
• The Abstract uses several parentheticals and statistics that disrupt flow; simplifying the presentation will enhance clarity.
• Terms like “clinician-level” need clearer operational definition—does this refer to average clinician performance, best clinician, or a panel?
• P-values should be supported by effect sizes or confidence intervals where applicable.
• The statement "diagnosis-driven matches at top ranks" may be unclear to clinical readers—consider rephrasing or explaining the notation.
• Expand on clinician feedback mechanisms—was it structured, validated, or open-ended?
The manuscript presents a high-quality, translational application of ML in paediatric cardiology with demonstrable clinical impact. However, key clarifications are needed in methodology, transparency, and evaluation metrics to support the robustness and reproducibility of findings. Additionally, improvements in explanation, clinical contextualization, and ethical framing will help broaden its appeal and utility.
Recommendation: Major revision

(Remarks on code availability)
N/A

Reviewer #6

(Remarks to the Author)
This article as a whole is a very comprehensive study, ranging from model development to clinical evaluation in various scenarios. However, from a technical perspective, no new methods or technologies have been proposed. At the same time, it can be said that many of the methods are not optimal, and the author may lack a systematic review of the relevant technologies in the field. However, the merit of this research lies in the in-depth clinical application research it has carried out. Of course, there are still many unclear points in this that require further explanation by the author. The following are some specific questions:

1.In the background introduction section, the author might have overlooked a very relevant study.

Reference: A Patient Similarity Network (CHDmap) to Predict Outcomes After Congenital Heart Surgery: Development and Validation Study. JMIR Med Inform 2024 Jan 19:12:e49138. doi: 10.2196/49138.
That study was also aimed at CHD, based on patient similarity analysis as well, and LOS was also one of the predictive indicators.

2. When introducing patient similarity analysis, an important stage of development was overlooked. Before deep learning, there were already excellent research cases on patient similarity analysis.
A patient-similarity-based model for diagnostic prediction. International Journal of Medical Informatics 2020,135 104073.

3.In the study, there is a lack of some basis for the classification of LOS, short-stay(1-3days) and long-stay(4+ days). As far as I know, for CHD surgery, the usual LOS is usually greater than 4 days. Is such a classification not suitable for such scenarios?
It is suggested to add the specific information of LOS in Table 1, such as the specific quantity distribution of short-stay and long-stay. This data is also beneficial for readers to understand why class imbalance and upsampling occur.

Based on the Supplementary Table S2-5, The indicators of Long Stay are significantly better than those of Short Stay, which to some extent indicates that the current classification is not very effective.

4.Furthermore, it is necessary to clarify whether 5-fold cross-validation occurs on the data after upsampling or whether upsampling only happens on the training data after the 5-fold data partitioning.

5. In Table 2 “All metrics were computed on an independent test set (MIMIC-IV; n=12,319, GOSH; n=301)” , for this study , the primary goal is focus on the pediatric cardiology, so it is more reasonable to only show the results on the GOSH n=301. The abundance of MIMIC-IV data might have masked its performance at GOSH cohort.

6. Regarding the calculation of ICD-10 diagnostic similarity, the use of character matching is not reasonable. First of all, different disease classifications may share the initial letters, such as D48 and D50, H59 and H60; Many studies have provided better methods for calculating the similarity of ICD-10 diagnoses and how to perform better similarity calculations on the diagnostic list.
Ref:
Using the distance between sets of hierarchical taxonomic clinical concepts to measure patient similarity. BMC Medical Informatics and Decision Making. 2019 19:91.

7. Regarding the introduction of the clustering situation in Table4, there are some points that need to be explained. The total data volume of the three clusters is 369. Which group of people does this correspond to? It is not the 301 validation dataset.

8.The values of the Mortality Rate in Supplementary Table 8, which are 20% and 40%, seem a bit high. Could specific values for the Mortality Rate of the entire population be given in Table1?

9.In the Silent Deployment within GOSH section, the reference Table for demopgrahoic data is incorrect. It should be Supplementary Table S7.

10.In the perfomance section of 3.3 Slient deployment, the RFmodel was deployed to GOSH to handle six months of real data. Why is there a 5k-validation fold? Theoretically, once this model is deployed, it can only provide one verification result. In addition, please provide the specific statistical data of Short stays and Long stays during this six-month period.

11. Are the members of the clinical panel for the clinical assessment from GOSH and the corresponding departments? There might be situations here, such as whether these cases are historical ones they have handled. Or, if they are not clinical staff from the corresponding department at GOSH, on what basis do they make their predictions?

12.Table S4 mentioned in the title of Table 4 on page 13 should be Table S8. In addition, the figures in Mortality in the table are not consistent with those in Figure 4b. The accuracy of the data needs to be checked again.

13. Figure 9 on page 14 should be Supplementary Figure S5.

14. It is not clear how The author got "The mean scores indicate that the model offers insights that extend beyond those a clinician would have considered at the time of consultation without presenting a lot of redundant or irrelevant information”. Doesn't the fact that the Utility Score is only 4 indicate that users are not very satisfied with this tool?

15. In the discussion section, the explanation for the poor performance of Short Stay was mentioned, suggesting that "predicting shorter stays is inherently more complex due to variability". It seems that the impact of Mortality has not been discussed. There is a relatively high Mortality rate in the dataset. It is unclear whether these patients who died within 3 days were included in the prediction of short stay. Then there may be two extreme situations among them. One type is discharged from the hospital within three days due to a mild condition, and the other type dies due to a critical condition. From a clinical perspective, this trial assessment included too many variables. Perhaps it should be limited to a relatively small scope, such as only evaluating patients who underwent CHD surgery, while classifying deceased patients separately.

16. There are some errors in the citations, such as citation 12 "Sun Y, Song X, Zhang y. Deep Dynamic Patient Similarity Analysis: Model Development and Validation in ICU. Computer Methods and Programs in Biomedicine. 2022; 225:107033. The author's information is incorrect.

(Remarks on code availability)
There is no code available for this paper. But I prefer the author can share it.

Version 1:

Reviewer comments:

Reviewer #5

(Remarks to the Author)
Thanks for the edits..good to go now!

(Remarks on code availability)
All edits I requested are done!

Reviewer #6

(Remarks to the Author)
Thank you, author, for answering almost all my questions seriously. I have no further questions. In addition, the author information of one of the citations is still incorrect. Please check and confirm the author information of citation 14.

(Remarks on code availability)
Due to the privacy of private data, this code is based on the open MIMIC dataset, making this research repeatable.

Open Access This Peer Review File is licensed under a Creative Commons Attribution 4.0 International License, which permits use, sharing, adaptation, distribution and reproduction in any medium or format, as long as you give appropriate credit to the original author(s) and the source, provide a link to the Creative Commons license, and indicate if changes were made.
In cases where reviewers are anonymous, credit should be given to 'Anonymous Referee' and the source.
The images or other third party material in this Peer Review File are included in the article's Creative Commons license, unless indicated otherwise in a credit line to the material. If material is not included in the article's Creative Commons license and your intended use is not permitted by statutory regulation or exceeds the permitted use, you will need to obtain permission directly from the copyright holder.
To view a copy of this license, visit https://creativecommons.org/licenses/by/4.0/

Reviewer Response

We would like to thank the reviewers for their time and insightful feedback. We have carefully revised the manuscript in line with their suggestions and made updates where appropriate. In this letter, we provide a detailed response to each reviewer's comment and outline the corresponding changes made to the manuscript, which are highlighted in blue.

1. Reviewer #5

Thank you for the opportunity to review this manuscript. The authors present a timely and technically ambitious study leveraging machine learning (ML) methods for predicting length of hospital stay (LoS) and retrieving similar patient cases in paediatric cardiology. This area of research is particularly valuable given the complexity and heterogeneity of congenital heart disease and other pediatric cardiac conditions. The manuscript demonstrates solid innovation and implementation rigor, though there are areas that require further clarification, refinement, and discussion before publication.

Narrative Structure, Grammar, and Flow

The manuscript is generally well-written and adheres to standard academic structure. The flow of ideas is logical, with a smooth transition between sections. However, some sentences in the Abstract and Discussion sections are densely packed with multiple technical elements and could benefit from clearer segmentation for reader comprehension. Additionally, a few instances of passive voice and overly complex phrasing may hinder clarity for multidisciplinary audiences.

1.1 Introduction

The introduction clearly outlines the motivation for applying ML in paediatric cardiology, where clinical complexity, data sparsity, and unpredictability in disease trajectories pose significant challenges. However, the current version would benefit from:

1.1.1 A deeper review of previous work on ML for LoS prediction and patient similarity in paediatric or adult populations, including any limitations.

We have discussed the limitations of previous works within the introduction/background section. We have revised the section, and an additional three studies have been included in the LoS and patient similarity sections (Daghistani *et al*. (2019), Jia *et al*. (2020), and Li *et al*. (2024)), offering further insights into the methodologies previously used in LoS predictive studies and patient similarity settings (Introduction, page 2, paragraphs 2 and 4).

1.1.2 An explicit gap statement explaining what this study does that prior studies did not—especially in terms of real-world clinical deployment.

We have included an explicit gap statement in the last paragraph of the introduction. (*"There remains an unmet need in the literature for research on patient similarity in paediatrics. Moreover, the heterogeneity of rare and complex disease introduces an additional layer of difficulty for developing such models.",* Introduction, page 3, paragraph 2)

1.2 Rationale and Novelty

The integration of LoS prediction with case similarity retrieval, and benchmarking against clinician performance, reflects commendable novelty. Silent deployment within the NHS and pilot feedback from clinicians further enhance translational relevance. This dual utility (predictive + retrieval) framework is innovative, though the underlying conceptual link between these two tasks could be made more explicit in the Methods and Discussion.

1.3 Methodology

The methodological pipeline is broadly appropriate and adheres to contemporary standards in clinical ML studies. Strengths include:

- Use of BioClinical-BERT for semantic embedding extraction.
- External comparison with clinicians as a reference standard.
- Real-world deployment in a large NHS system.

However, several methodological concerns need clarification:

1.3.1 Data Quality & Missingness: There is no detailed account of missing data handling, imputation strategies, or data completeness across EHR variables. Were time-series features complete for all 1,002 patients?

We thank the reviewer for highlighting this. EHR records are collected as part of routine clinical care, and in any real-world setting, they are subject to missing values. In this work, the primary focus was on using ICD codes, medications, laboratory results, and procedures. As these data types are closely monitored within the hospital, they are generally more complete relative to other EHR components, such as vital signs and other elements that might have missing values. Additionally, the data has been audited by the hospital, ensuring a high level of completeness and reliability.

The two modelling approaches applied in the study are capable of handling the different data structures. LLMs can inherently handle variable data lengths, while the Random Forest model treats missing data as indicative of the absence of information or diagnosis. To minimise bias, we did not perform data imputation. It was important that the models reflect the realities of the routinely collected data and care provided in a hospital setting rather than rely on imputed values. Rather than imputing to improve performance metrics, we aimed to ensure modelling reflected the realities of clinical care.

1.3.2 Feature Engineering: It is unclear which structured or unstructured features were used, how they were processed (e.g., one-hot encoding, normalization), and whether any selection techniques were applied.

Thank you for this question. We have added further clarification to the methodology section (Methods, Sections 4.2 and 4.3, page 13). The LLM receives the data as a string, representing the events listed that occurred on the day of admission. For the Random Forest model, the same string is used; however, it is processed using a "Count Vectorizer", which transforms the text into a numerical representation based on the frequency of each event. Standard one-hot encoding was not used, as it would have resulted in a very large feature space. Additionally, specific ICD codes often co-occur, which could lead to high multicollinearity and potential loss of information.

1.3.3 Timeline Alignment: The process of aligning patient records to a 24-hour timeline is mentioned briefly. This step warrants a fuller description—**what is the unit of aggregation**? How was **temporal sparsity** handled?

Thank you for this question. In consultation with the clinical experts involved in this study, we identified an important practical consideration for data preprocessing. The timestamp for ICD codes documented within the EHR system does not necessarily reflect exactly when the event occurred.

Similarly, administering a treatment is not necessarily recorded at the exact time of administration. For example, a diagnosis code may be entered hours or even days after the condition is first identified, and medication administration times may differ from their documentation timestamp.

To ensure temporal consistency and accuracy, all events were aggregated on a 24-hour time frame. The LoS labels were defined using the same unit. The data follows a time course (not even distribution), and there might be a scarcity. Aggregation on a daily basis provides a reliable representation of when medical events occurred. This provides further confidence in the accuracy of the similarity search.

1.3.4 Model Interpretability: While RF models are generally interpretable, there is no report of **feature importance, SHAP values,** or clinical reasoning correlates—key for clinical trust

Thank you for your comment. We acknowledge the importance of model interpretability, especially in clinical settings. The corresponding feature importance analysis is included in Appendix Supplementary Figure 1. Due to the word count constraints of *Nature Communications,* we placed the feature importance in the appendix and discussed the important features within the text.

1.3.5 Similarity Retrieval Evaluation: How was the retrieval performance quantified beyond anecdotal clinician feedback? Was there any ranking metric like MRR, NDCG, or qualitative rating scale applied to top-k matches?

Thank you for highlighting this. This comparison was conducted in two parts. The first part evaluates qualitative metrics, described in Section 4.5.1, and retrieval@ *n* performance based on cosine similarity, hierarchical similarity mappings and categorical similarities, with results presented in Table 5 (Section 2.4). The second part involves a clinical evaluation with six clinicians, and the corresponding results are reported in Table 7.

1.3.6 Figures and Tables

Ensure clear and labelled confusion matrices or ROC curves for LoS prediction.

We have added the confusion matrices for the best-performing LLM-based model (BioClinical BERT MIMIC-GOSH) and the best-performing overall model (Random Forest) to the Appendix. The results are included in Supplementary Table 6.

Include representative retrieval results or embedding cluster visualisations (e.g., t-SNE/UMAP).

We agree that it is important to include the visualisations. We would like to draw attention to Figure 4a in the Results. This figure shows the three-dimensional t-SNE of the embeddings. Table 4 also shows the patient profiling of the identified clusters represented in Figure 4a.

Consider adding clinician-level performance variability and model confidence stratification.

To provide stronger support in the decision-making capabilities of the clinical tool, we provide a traffic light system stratified according to confidence intervals. We have updated the manuscript accordingly (Section 4.5, page 14). The results can be found in Supplementary Table 7. This method aims to maximise the Youden's J index, consequently increasing specificity and slightly decreasing sensitivity to reduce the models' slight bias towards longer LoS.

Tables should compare model metrics vs. clinician estimates over the same cohort.

Both the panel of clinicians and the model received the same cohort of patients. Therefore, Figure 2 and Table 4 show comparable values between the same patient cohort. We have revised the text and included further details in the figure caption to provide the reader with further clarity (Figure 2 caption, page 6).

1.4 The results are compelling, particularly the LoS prediction performance (accuracy 0.91 ± 0.01) and the reported superiority over clinicians ($p < 0.001$). Additional strengths include:

- High precision and sensitivity metrics across six months of silent deployment.
- Identification of three clinically distinct subgroups through unsupervised clustering.
- Inclusion of subjective clinician feedback in real clinical settings.

However, results could be strengthened by:

- Disaggregating model performance by key patient subgroups (age, primary diagnosis, comorbidities).

Subgroup analyses of model performance by sex and ethnicity were conducted; however, due to the word count restrictions, these results are presented in the Appendix (please refer to Supplementary Tables 2-5). Due to the high diversity of primary diagnoses and comorbidities (reflecting the nature of rare and complex paediatric conditions at this centre), the sample size of each group is small for individual subgroups. As such, disaggregation was not conducted as it would limit the statistical validity of such analyses.

In terms of reporting misclassification rates and common sources of error. We have revised the text and included confusion matrices in the appendix (please refer to letter Point 1.3.6). The confusion matrices provide the reader with further understanding of the misclassification rate for the best-performing model (RF) and the best-performing LLM-based model (BioClinical BERT MIMIC-GOSH).

- Providing comparative performance of all tested models – not just RF.

We have revised the text and provided comparative performance results for all tested models on the retrospective dataset (please refer to Tables 1 and 2). For prospective data, we ran the pre-trained model to assess the method's utility using unseen data in the operational environment. For the pilot study, we selected the best-performing model. Our aim was to evaluate whether this model would maintain its performance when deployed in a real-world clinical setting.

1.5 The Discussion appropriately highlights the clinical relevance and future potential of ML-based prediction and retrieval systems. Nonetheless, a few enhancements are needed:

- Limitations: The authors do not fully engage with limitations such as generalizability beyond GOSH, algorithmic bias in small datasets, or the risks of overfitting in niche populations.

We have revised the text and added additional information to the discussion section regarding the above-mentioned points (Discussion, page 12, paragraph 4).

- Ethical Considerations: There is no mention of ethical oversight, potential for automation bias, or plans for integrating human–AI collaboration in decision-making.

While the tools created provide values for LoS, the primary goal is to create a system to work in collaboration with clinical experts. The aim of the model and similarity retrieval is not to make decisions autonomously but to offer contextual information to enhance, rather than automate, clinical judgments. As such, we provide a system that functions as a clinical decision support tool, mitigating automation bias and allowing for the final decision to remain under the supervision of the clinician (Discussion, page 12, paragraph 4).

- Scalability: How transferable is the model to other NHS settings or international hospitals with different EHR systems?

The model is inherently transferable and scalable across different hospital settings, as its inputs are based on standardised data sources, including ICD codes, procedure codes (OPCS system), medication codes, and commonly used laboratory tests. ICD codes and medication data are widely standardised, which minimises variation across institutions. Laboratory tests may exhibit some differences in measurement practices or reference ranges, but these differences can be addressed through further fine-tuning. As a result, the model can be transferred to other hospitals with

relatively minimal adaptation, requiring only limited additional fine-tuning to reflect local practices and ensure safety (Discussion, page 12, paragraph 4).

1.6 Other Minor Comments

- The Abstract uses several parentheticals and statistics that disrupt flow; simplifying the presentation will enhance clarity.

We acknowledge that the Abstract contains parentheticals and statistics that may disrupt readability. However, Nature's guidelines require that we report the significance of our findings. As such, a brief summary of the key findings and statistics is needed to report the significance to ensure that the results are fully interpretable on their own.

- Terms like "clinician-level" need clearer operational definition—does this refer to average clinician performance, best clinician, or a panel?

Thank you for this suggestion. We have revised this and changed from clinician-level to clinically applicable (Manuscript Title).

- P-values should be supported by effect sizes or confidence intervals where applicable.

Thank you for this suggestion. We have added effect sizes throughout Section 2.4. For mean LoS, Hedges' *g* was computed for each pairwise Welch t-test (Group 1 vs 2: g=0.49; Group 2 vs 3: g=0.41). For emergency and complication rates, we use Fisher's exact tests and report odds ratios with 95% confidence intervals (CI). Following reanalysis, a significant difference was observed between Groups 2 and 3 (p = 0.0124, odds ratio = 0.26, 95 % CI [0.09-0.74]). Figure 4b and the text in Section 2.4 have been updated accordingly.

- The statement "diagnosis-driven matches at top ranks" may be unclear to clinical readers—consider rephrasing or explaining the notation.

We have revised the wording in the second half of the Abstract to improve clarity. Specifically, the description now reads: "*Cosine similarity retrieval reveals diagnosis-driven top matches…*"

- Expand on clinician feedback mechanisms—was it structured, validated, or open-ended?

Due to page limitations, we have provided further details in the Appendix (Supplementary Figure 5). This feedback was collected in a structured manner, with clinicians required to provide data in a similar structure to the models' input data. The clinicians were required to state between 1-10 if the results were relevant.

2. Reviewer #6

This article as a whole is a very comprehensive study, ranging from model development to clinical evaluation in various scenarios. However, from a technical perspective, no new methods or technologies have been proposed. At the same time, it can be said that many of the methods are not optimal, and the author may lack a systematic review of the relevant technologies in the field. However, the merit of this research lies in the in-depth clinical application research it has carried out. Of course, there are still many unclear points in this that require further explanation by the author. The following are some specific questions:

2.1 In the background introduction section, the author might have overlooked a very relevant study. Reference: *A Patient Similarity Network (CHDmap) to Predict Outcomes After Congenital Heart Surgery: Development and Validation Study. JMIR Med Inform 2024 Jan 19:12:e49138. doi: 10.2196/49138.*
That study was also aimed at CHD, based on patient similarity analysis as well, and LOS was also one of the predictive indicators.

When introducing patient similarity analysis, an important stage of development was overlooked. Before deep learning, there were already excellent research cases on patient similarity analysis.
A patient-similarity-based model for diagnostic prediction. International Journal of Medical Informatics 2020,135 104073.

We thank the reviewer for drawing our attention to these two studies. Information about the two papers has been included in the background literature in the introduction section and cited accordingly (Introduction, page 2, paragraph 4).

2.2 In the study, there is a lack of some basis for the classification of LOS, short-stay(1-3days) and long-stay (4+ days). As far as I know, for CHD surgery, the usual LOS is usually greater than 4 days. Is such a classification not suitable for such scenarios?

To determine the optimal time boundaries for short and long hospital stays, the literature and clinicians at GOSH were consulted, as stated on page 4 of the paper. The aim of these discussions and the literature review was to identify the most appropriate threshold for bed management in a hospital setting. The study by Daghistani *et al.* (2019) specifically investigated LoS in the cardiology ward in the context of bed capacity, defining a short stay as up to 3 days. Clinicians at GOSH, however, suggested that due to the rare nature of many diseases treated there, this threshold should be extended by one day.

Overall, as GOSH is a centre for complex and rare diseases, the aim has been to identify whether using admission data and routinely collected healthcare data, we can reliably predict the short and long stays. The latter is most useful in hospital bed-management and also an indication of potential risk of complications.

2.3 It is suggested to add the specific information of LOS in Table 1, such as the specific quantity distribution of short-stay and long-stay. This data is also beneficial for readers to understand why class imbalance and upsampling occur.

Thank you for this very helpful suggestion. We have added two rows to Table 7 (Section 4.4, page 14) to present the distribution of short- and long-stay cases. This addition clarifies the imbalance between the two groups and helps readers better understand the rationale for the upsampling procedure applied in our analysis.

2.4 Based on the Supplementary Table S2-5, The indicators of Long Stay are significantly better than those of Short Stay, which to some extent indicates that the current classification is not very effective

Thank you for your comment. We agree that, as shown in Supplementary Tables S2–S5, the indicators for Long Stay patients are significantly better than those for Short Stay, which may suggest that the current classification has limitations. However, there are important clinical factors that help explain this pattern.

GOSH is a specialist centre for complex and rare diseases. The hospital acts as a national referral hub for these cases and performs over 100 paediatric heart transplants per year. Due to the complex nature of many of these conditions, longer lengths of stay (LoS) are often more predictable. In clinical practice, variation and heterogeneity are more likely to occur when a patient presents with a condition that would usually result in a short stay, but due to comorbidities or complications, they stay longer than expected. This unpredictability makes Short Stay cases harder for the model to classify correctly.

We agree that longer stays tend to exhibit more consistent patterns, which the model is better able to learn and predict. While the model performs worse on Short Stay cases, it still achieves 72% accuracy, demonstrating useful predictive power. This also reinforces the value of our model's multifaceted prediction approach, which incorporates patient similarity to highlight cases where an adverse event or hidden risk may lead to an extended stay. We have revised the text to reflect these aspects (Discussion, page 11, paragraph 2).

2.5 Furthermore, it is necessary to clarify whether 5-fold cross-validation occurs on the data after upsampling or whether upsampling only happens on the training data after the 5-fold data partitioning.

Upsampling with replacement was applied only to the train set within each fold. To prevent data leakage, we ensured that the upsampled data did not appear in both the training and validation sets. To provide further clarity to the reader, further explanation has been added to the methods (Section 4.3, page 13).

2.6 In Table 2 "All metrics were computed on an independent test set (MIMIC-IV; n=12,319, GOSH; n=301)" , for this study, the primary goal is focus on the paediatric cardiology, so it is more reasonable to only show the results on the GOSH n=301. The abundance of MIMIC-IV data might have masked its performance at GOSH cohort.

We thank the reviewer for this comment. We have broken Table 2 into Table 1 and 2 (Section 2.1, page 5) to provide stronger clarity to the reader. We provide information about model performance with MIMIC data due to the private nature of the data from GOSH. The MIMIC data assists in reproducibility of the experiments.

2.7 Regarding the calculation of ICD-10 diagnostic similarity, the use of character matching is not reasonable. First of all, different disease classifications may share the initial letters, such as D48 and D50, H59 and H60; Many studies have provided better methods for calculating the similarity of ICD-10 diagnoses and how to perform better similarity calculations on the diagnostic list.

Ref:
Using the distance between sets of hierarchical taxonomic clinical concepts to measure patient similarity. BMC Medical Informatics and Decision Making. 2019 19:91.

We appreciate the reviewer's thoughtful feedback regarding ICD-10 similarity calculations. In our initial approach, we used full ICD-10 codes to preserve diagnostic specificity, but we recognise that this method may not fully capture the hierarchical relationships between codes, which are often clinically meaningful.

In response to the reviewer's suggestion, we have incorporated the method described in *"Using the distance between sets of hierarchical taxonomic clinical concepts to measure patient similarity"* (BMC Medical Informatics and Decision Making, 2019) into our analysis. This approach allows for a more nuanced similarity calculation and has been described in Section 4.5.1 of the manuscript. Table 5 (Section 2.4, page 9) has also been updated to reflect the results of this additional similarity analysis. We have clarified the original implementation to avoid confusion and appreciate the opportunity to strengthen this aspect of the study.

2.8 Regarding the introduction of the clustering situation in Table4, there are some points that need to be explained. The total data volume of the three clusters is 369. Which group of people does this correspond to? It is not the 301 validation dataset.

We thank the reviewer for pointing out these inconsistencies. The cluster population size of 369 is correct; however, the initial demographics table was based on an outdated version of the dataset. We have now updated it accordingly — the training/validation set includes 1,524 patients, and the test set includes 382 patients. All demographic information in Table 7 (Section 4.4, page 14) has been corrected to reflect these revisions.

The discrepancy between the test set size (n = 382) and the number of patients included in the clustering analysis (n = 369) arises because 13 patients lacked primary diagnosis information. As the clustering analysis focused on primary diagnoses, these patients were excluded from that specific analysis. We have revised the text in Section 4.5.2 (page 16) of the paper to provide further clarity about the clustering cohort size.

2.9 The values of the Mortality Rate in Supplementary Table 8, which are 20% and 40%, seem a bit high. Could specific values for the Mortality Rate of the entire population be given in Table1?

We thank the reviewer for drawing our attention to this point. The labelling of the y-axis in Figure 4b was misleading. The 20% and 40% figures do not represent the overall mortality rate, but rather the proportion of all deaths within the clustering cohort that fall into each category.

We have revised the axis labelling to provide clarity. The mortality rate of each individual cluster cohort can be found in Supplementary Table 8. We have changed the death proportion column to mortality rate to provide this information to the reader. The mortality values are not higher than expected, especially when considering the specialised nature of GOSH. The overall mortality rate of the train and test sets of the GOSH and MIMIC data has been included in Table 1 (Section 4.4, page 14).

2.10 In the Silent Deployment within GOSH section, the reference Table for demographic data is incorrect. It should be Supplementary Table S7.

Thank you for drawing our attention to this. The Supplementary Table reference has been updated to correct this issue.

2.11 In the performance section of 3.3 Silent deployment, the RFmodel was deployed to GOSH to handle six months of real data. Why is there a 5k-validation fold? Theoretically, once this model is deployed, it can only provide one verification result.
In addition, please provide the specific statistical data of Short stays and Long stays during this six-month period.

We thank the reviewer for noticing this. Stating five-fold validation is incorrect in this setting. We have corrected it to "distribution of recall values" in both the Figure 3 and the Supplementary Figure 3 caption (pages 7 and 28 respectively).

Regarding the specific statistical data for the short and long stays, we have added this into Supplementary Table 9.

2.12 Are the members of the clinical panel for the clinical assessment from GOSH and the corresponding departments? There might be situations here, such as whether these cases are historical ones they have handled. Or, if they are not clinical staff from the corresponding department at GOSH, on what basis do they make their predictions?

The majority of the clinical panel members were from GOSH. However, as the data was anonymised and the large quantity of patients a clinician sees, it is highly unlikely that confirmation bias has occurred. In the silent deployment and pilot study, prospective data were used, and the clinicians were asked to predict the outcome before the outcome had happened.

2.13 Table S4 mentioned in the title of Table 4 on page 13 should be Table S8. In addition, the figures in Mortality in the table are not consistent with those in Figure 4b. The accuracy of the data needs to be checked again.

We thank the reviewer for drawing our attention to this point. We have corrected the figure, as the y-axis should not have claimed ‘rate (percentage)’ but the proportion of overall deaths/emergency/complications per cluster (Figure 4b, Section 2.4, page 8).

The numbers in the Supplementary Table 10 have been updated to include information regarding the rates, not proportions, of the different categories, providing additional information to the reader.

2.14 Figure 9 on page 14 should be Supplementary Figure S5.

Thank you for highlighting this. This point has been addressed and corrected in the manuscript.

2.15 It is not clear how the author got "The mean scores indicate that the model offers insights that extend beyond those a clinician would have considered at the time of consultation without presenting a lot of redundant or irrelevant information”. Doesn't the fact that the Utility Score is only 4 indicate that users are not very satisfied with this tool?

In the questionnaire, we aimed to discover how much clinicians find the provided similarities insightful, particularly in revealing insights they might not consider during routine consultations. Since the cardiology unit is highly specialised, the model identifies both expected similar cases and nuanced patterns to be highlighted through similar cases to the clinicians. Regarding the latter, a very high score of insight utility was not anticipated (as it would suggest the tool offers more confirmation bias), and a very low score would indicate little to no utility. As discussed in the section (page 12, paragraph 2), we expected only a subset of similar cases to offer higher insights and utility, which is reflected in the overall scores. Throughout the paper, working closely with experts involved in bedside care and consultation, our goal has been to develop a tool that provides the most effective insights and utility without overwhelming clinicians with information (which would not be practical in clinical settings).

The tool is capable of providing information that the clinicians are not already aware of. Therefore, the score indicates that the model can be used in a complementary role alongside the existing decision-making skills of the clinicians. This underscores that the tool is not intended to replace clinical expertise but to strengthen and extend it.

2.16 In the discussion section, the explanation for the poor performance of Short Stay was mentioned, suggesting that "predicting shorter stays is inherently more complex due to variability". It seems that the impact of Mortality has not been discussed. There is a relatively high Mortality rate in the dataset. It is unclear whether these patients who died within 3 days were included in the prediction of short stay. Then there may be two extreme situations among them. One type is discharged from

the hospital within three days due to a mild condition, and the other type dies due to a critical condition. From a clinical perspective, this trial assessment included too many variables. Perhaps it should be limited to a relatively small scope, such as only evaluating patients who underwent CHD surgery, while classifying deceased patients separately.

Thank you for highlighting this. As we also discussed in the reviewer's comment in 2.13, as the mortality was incorrectly labelled, this resolves the issues mentioned above, as the mortality rate is no longer considered relatively high. If the mortality rate is slightly higher at GOSH when compared to other hospitals, this is due to the highly specialised nature of the hospital, placing specific emphasis on rare diseases.

2.17 There are some errors in the citations, such as citation 12 "Sun Y, Song X, Zhang y. Deep Dynamic Patient Similarity Analysis: Model Development and Validation in ICU. Computer Methods and Programs in Biomedicine. 2022; 225:107033. The author's information is incorrect.

We thank the reviewer for noticing these errors and have corrected them accordingly in the paper.

2.18 Code availability

We have uploaded the code to GitHub (https://github.com/tmi-lab/GOSH-EHR-cardiology).

We would like to thank the reviewers once again for their time and valuable comments, which have contributed to the improvement of this work.

Yours sincerely,

Payam Barnaghi
(on behalf of all authors)

References

Daghistani, T.A., Elshawi, R., Sakr, S., Ahmed, A.M., Al-Thwayee, A. and Al-Mallah, M.H. (2019). Predictors of in-hospital length of stay among cardiac patients: A machine learning approach. *International Journal of Cardiology*, 288, pp.140–147. doi:https://doi.org/10.1016/j.ijcard.2019.01.046.

Jia, Z., Lu, X., Duan, H. and Li, H. (2019). Using the distance between sets of hierarchical taxonomic clinical concepts to measure patient similarity. *BMC Medical Informatics and Decision Making*, 19(1). doi:https://doi.org/10.1186/s12911-019-0807-y.

Jia, Z., Zeng, X., Duan, H., Lu, X. and Li, H. (2020). A patient-similarity-based model for diagnostic prediction. *International Journal of Medical Informatics*, [online] 135, p.104073. doi:https://doi.org/10.1016/j.ijmedinf.2019.104073.

Li, H., Zhou, M., Sun, Y., Yang, J., Zeng, X., Qiu, Y., Xia, Y., Zheng, Z., Yu, J., Feng, Y., Shi, Z., Huang, T., Tan, L., Lin, R., Li, J., Fan, X., Ye, J., Duan, H., Shi, S. and Shu, Q. (2024). A Patient Similarity Network (CHDmap) to Predict Outcomes After Congenital Heart Surgery: Development and Validation Study. *JMIR Medical Informatics*, 12, pp.e49138–e49138. doi:https://doi.org/10.2196/49138.

Reviewer Response (January 2026)

Reviewer #6 (Remarks to the Author):

Thank you, author, for answering almost all my questions seriously. I have no further questions. In addition, the author information of one of the citations is still incorrect. Please check and confirm the author information of citation 14.

Thank you for highighting this. Citation 14 has now been corrected:

Sun Z, Lu X, Duan H, Li H. Deep Dynamic Patient Similarity Analysis: Model Development and Validation in ICU. Computer Methods and Programs in Biomedicine. 2022;225:107033.
